# Interaction of artificial intelligence, mental disorders, and diverse data modalities: Potential treatment management based on the “method–disease–data” axis

**DOI:** 10.4103/NRR.NRR-D-25-00121

**Published:** 2025-09-03

**Authors:** Xu Tian, Ning Wang, Jin Yan, Yiming Chen, Ke Ma

**Affiliations:** 1 Guangdong Provincial Key Laboratory of Food, Nutrition and Health, Department of Toxicology, School of Public Health, Sun Yat-sen University, Guangzhou, Guangdong Province, China; 2 Shandong Co-Innovation Center of Classic TCM formula, Shandong University of Traditional Chinese Medicine, Jinan, Shandong Province, China; 3 Office of Academic Research, Shandong University of Traditional Chinese Medicine, Jinan, Shandong Province, China; 4 Department of Rehabilitation Medicine, Ruijin Hospital, Shanghai Jiao Tong University School of Medicine, Shanghai, China; 5 Department of Acupuncture-Moxibustion and Tuina, Shandong University of Traditional Chinese Medicine, Jinan, Shandong Province, China; 6 School of Medicine, Shandong University of Traditional Chinese Medicine, Jinan, Shandong Province, China

**Keywords:** artificial intelligence, clinical decision support, clinical trial, computational psychiatry, early diagnosis, knowledge discovery, personalized medicine, precision psychiatry, psychiatric disorders, treatment prediction

## Abstract

Although many previous studies have highlighted the advances in prediction models, instruments for pathological and histological diagnosis and treatment, as well as individualized treatment modalities in mental disorders, these previous syntheses usually study the research outcomes separately and ignore the holistic integration of research regarding artificial intelligence technological approaches, data sets used and applications in mental health research. We used the BioBERT pretrained language model to systematically extract relevant information and develop an extensive knowledge graph that includes 3158 entities connected with 3248 different relationships. Our knowledge graph delineates essential artificial intelligence technological frameworks and explicitly maps out the relationships linking artificial intelligence methods, mental disorders, and diverse data modalities. The synthesis, centered on the analytical axis of “method-disease-data,” highlights key research areas where artificial intelligence and neuropsychiatry meet. Specifically, it focuses on key applications in early detection, improved accuracy of diagnosis, and individualized therapeutic interventions. In addition, we summarized the applications derived from basic research findings that extend to ongoing clinical trials, revealing the path toward future clinical application in psychiatric work. Importantly, the research paid special attention to the application of artificial intelligence in identifying key brain regions and neural circuits, providing important clues for elucidating the neural mechanisms of mental disorders and developing targeted interventions. Although artificial intelligence presents great opportunities, there are also significant challenges, including imbalanced data sets, ethical issues, and clinical concerns about trustworthiness and transparency. Strategies to address these challenges are proposed, and a perspective on emerging methods enabled by artificial intelligence is provided, which are expected to greatly change the management and treatment of the future.

## Introduction

Artificial intelligence (AI) has been increasingly integrated into biomedical and life sciences research since the 1970s (Beam and Kohane, 2016). Despite some concerns, the integration of multimodal data from clinical and laboratory with continuous advancements in machine learning (ML) and deep learning (DL) algorithms, combined with enhanced computational capabilities and the integration of multimodal clinical and laboratory data, have enabled significant breakthroughs across various domains. These advancements notably include personalized medical diagnostics, identification of drug targets, and improvements in medical imaging techniques (Dilsizian and Siegel, 2014). Recently, the concept of “AI for Science” has emerged, representing a transformative frontier in global AI research. This initiative is accelerating shifts in traditional scientific methodologies, restructuring research paradigms, and contributing to a broader technological evolution (Taddeo and Floridi, 2018; Kanza et al., 2021; Berens et al., 2023). The advent of large language models (LLMs), such as AlphaFold (Abramson et al., 2024) for protein structure prediction, BioBERT (Lee et al., 2020) for biomedical text mining, and versatile models such as GPT (Brown et al., 2020) or MedPaLM (Singhal et al., 2025) specifically designed for medical question-answering tasks, has introduced new possibilities in the medical and health domains (Karabacak and Margetis, 2023; Lin, 2024).

Although the application of AI is becoming increasingly prevalent in the medical field related to physical health, it faces much more difficult and complex situations in the field of mental disorders (Jiang et al., 2017; Abondio and Bruno, 2025). Mental disorders exhibit complex pathophysiology and diverse clinical presentations, making diagnosis challenging and treatment outcomes uncertain (Blokhin et al., 2020). These disorders include conditions such as depression, bipolar disorder (BD), post-traumatic stress disorder (PTSD), autism spectrum disorder (ASD), and schizophrenia (SCZ), spanning interdisciplinary research from psychological sciences to investigation of neural mechanisms (Krystal and State, 2014; Cai et al., 2024). Currently, diagnostic methods still rely heavily on clinical experience rather than definitive biomarkers, complicating accurate prognostic assessment (Pilgrim, 2013). The traditional assessment methods based on Diagnostic and Statistical Manual of Mental Disorders, Fifth Edition (DSM-5) (American Psychiatric Association, 2013) and The International Statistical Classification of Diseases and Related Health Problems 11^th^ Revision (ICD-11) (World Health Organization, 2024) diagnostic criteria are being challenged by increasing evidence showing large pathophysiological heterogeneity among patients with the same diagnosis from these two sets of criteria. These results highlight the limitations of traditional assessment approaches, which often exhibit strong subjectivity and are not well adapted to the complexity and pathogenesis of psychiatric phenotypes. These limitations may lead to inaccurate diagnoses and suboptimal treatment approaches, potentially impacting clinical outcomes. Diagnostic approaches, treatment plans, and care models should move toward greater personalization and objectivity to address these challenges (Jiang et al., 2017; Karabacak and Margetis, 2023). Additionally, mental disorders are not limited to neurobiology, but are influenced by a complex interplay between other factors, including environmental exposures, psychosocial contexts, cognitive characteristics and genetic or epigenetic factors (van den Bosch and Meyer-Lindenberg, 2019; Zhao et al., 2024a). To have a comprehensive understanding of mental disorders, systematic integration of multimodal data such as neuroimaging, molecular profiles, behavioral metrics and clinical histories is required (Cole and Franke, 2017). However, the relationships between these levels are seldom one-to-one. A single biological abnormality may affect multiple psychological or cognitive functions, and conversely, similar symptoms may arise from totally different underlying conditions (Maia and Cano-Colino, 2015). This multidimensional complexity calls for the implementation of objective, data-integrated analytical frameworks that can synthesize diverse contributing elements and multimodal datasets.

ML and DL methods such as support vector machines (SVM), random forests (RF), neural networks, and Transformer models offer game-changing potential for mental health professionals to decode complex patterns across multidimensional datasets ranging from neurobiological markers, behavior, to genomics (Hyman, 2007; American Psychiatric Association, 2013; Insel and Cuthbert, 2015; World Health Organization, 2024). Many researchers have applied AI to process clinical records as well as ancillary data sources and convert heterogeneous inputs into vector spaces that support multidimensional analyses of mental disorders. These methods enable systematic study of the complexities of intervention for mental disorders through diverse analytical lenses. In precision psychiatry paradigms, combining identifiable and measurable endophenotypic markers in conjunction with clearly symptomatic expression for proactive clinical interventions and personalized medications may reduce long-term morbidity by psychiatric disorders (Farias et al., 2021). Consequently, the capacity of AI to advance mental health understanding, improve diagnosis, inform treatment and guide prevention interventions shows meaningful progress (Bzdok and Meyer-Lindenberg, 2018).

In recent comprehensive reviews focused on the application of AI in mental health research, remarkable progress in predictive modeling techniques, diagnostic approaches and personalized therapeutic strategies have been reported (Joshi and Singh, 2024). Although these reviews integrate results from various studies, most reviews adopt an isolated review approach that critiques single research components and seldom examines relationships among significant discoveries (Siotia et al., 2023). This approach leads to fragmented understanding and inadequately integrates various AI methods, their respective data formats and various usage scenarios under different psychiatric conditions, which hinders a comprehensive understanding of AI deployment in mental health research and clinical implementation (Babkova et al., 2024). Furthermore, contemporary analytical surveys mostly focus on theoretical explorations and rarely integrate outcomes from therapeutic interventions that have been enhanced by AI. Although conceptual studies provide rich foundational knowledge, the lack of practice validation creates an imbalanced analytical framework that hinders the practical applicability of scholarly examinations in genuine healthcare environments (Tornero-Costa et al., 2023).

To bridge gaps in the literature, we performed data mining and knowledge extraction from studies that bridged the AI and mental disorders intersection. A knowledge graph (KG) (Singhal, 2012) was constructed to systematically analyze and integrate AI methods, application domains, mental disorder categories and respective data formats identified in academic papers. This KG provides an organized framework to demonstrate practical applications of AI across different mental health conditions and enhances understanding of contemporary research in this domain. Through mapping these connections, our investigation uncovers critical trends in applying ML and DL methods when processing multidimensional data sources. Building on existing theoretical foundations, we critically evaluate AI-powered clinical trials for mental disorders through multiple perspectives including research progress, demographic variables and temporal dimensions. The analysis further identifies promising opportunities for AI implementation in psychiatric care while addressing current limitations through proposed mitigation strategies. Our investigation also explores emerging innovations at the AI-mental health intersection and presents a strategic framework for facilitating cross-disciplinary investigations. This research not only extends previous scholarly examinations but also provides new perspectives to guide future AI applications in mental health research and therapy.

**Figure 1:**
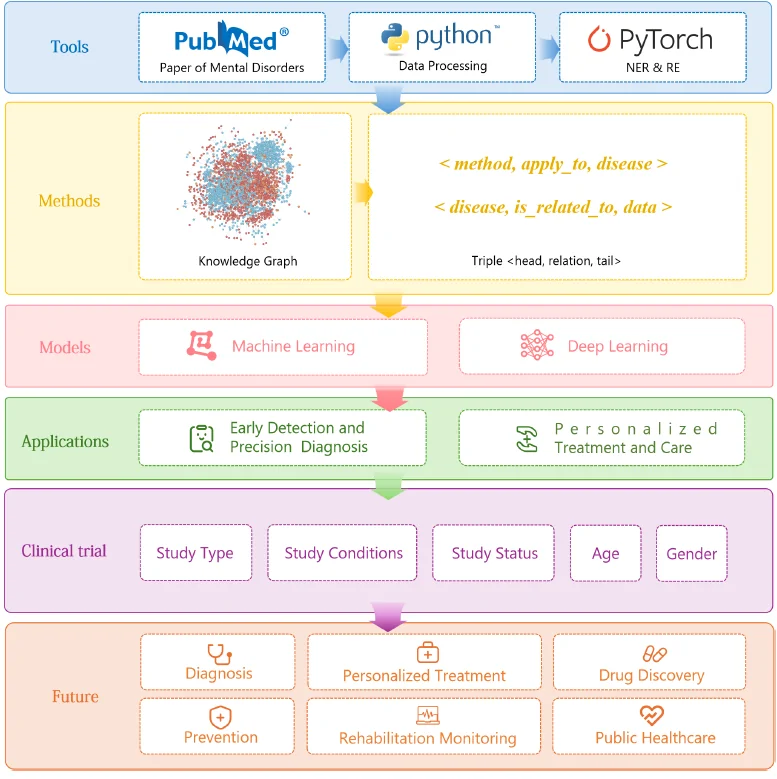
The workflow of this review. In this schematic, the overall process of literature mining, KG construction, AI model classification, application scenarios and clinical relevance analysis are illustrated. Relevant papers on mental disorders were mined from PubMed and further processed with Python and PyTorch. Then a KG was constructed to encode the relationships of <*method, apply_to, disease*> and <*disease, is_related_to, data*>. Such structured representations were further beneficial for classifying AI techniques (ML, DL) and their applications in early diagnosis and personalized treatment. Finally, clinical trial metadata, including study design, conditions, age and gender, were integrated to further improve the practical interpretation of model deployment. The lower panel shows possible future directions towards diagnosis, drug discovery, personalized medicine, prevention, rehabilitation monitoring and public mental healthcare. This figure includes icons from the IconPark open-source icon library (https://iconpark.oceanengine.com), used under the terms of the MIT License. AI: Artificial intelligence; DL: deep learning; KG: knowledge graph; ML: machine learning.

To provide a clearer understanding of our framework and methodological workflow, we briefly illustrate the workflow of this review in **Figure 1**. The figure shows the main processes of our approach, i.e., literature retrieval, KG construction, classification of AI methods, identification of applications, integration of clinical trials and forecasting future applications. This schematic also captures the layered relationships between data, models and psychiatric applications that this review carefully explores in subsequent sections.

## Spectrum of Artificial Intelligence

**Figure 2:**
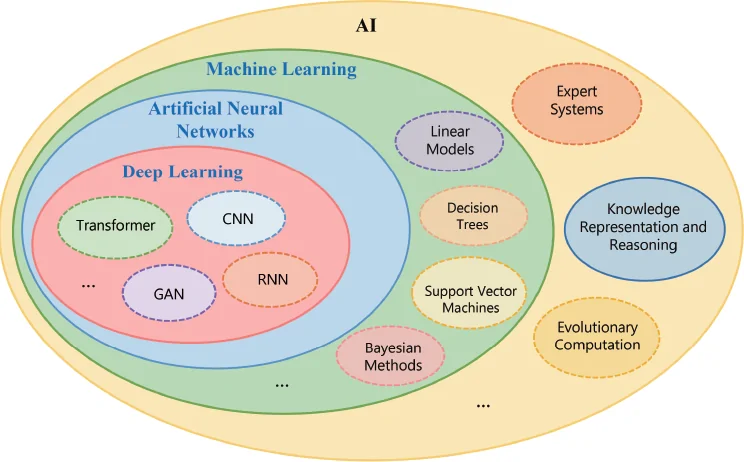
The spectrum of AI. This diagram illustrates the specific implementation methods involved in AI, highlighting the positions of machine learning and deep learning within the broader AI domain. It also depicts the relationships among these techniques, providing a concise framework of the technologies included. AI: Artificial intelligence; CNN: convolutional neural network; GAN: generative adversarial network; RNN: recurrent neural network.

AI represents a varied and complex domain of technological research aiming to replicate, enhance and expand human intelligence through conceptual framework, methodological approach, technological realization and application scenario (Tecuci, 2012). Its basic idea is to endow computational intelligence with anthropomorphic thinking abilities to reproduce human intelligence in sensory perception, reasoning ability in behavior execution, skill in perception and complex decision-making (Tripathi, 2021). AI takes the lead in developing autonomous systems that resemble human cognition and problem-solving. **Figure 2** shows the relationships between AI, ML, and DL in the technological ecosystem. Current technological solutions often combine these approaches by proper combination.

As a subset of AI, ML is an approach to develop adaptive algorithms and allow them to learn from data to improve their performance. Different from traditional systems with fixed rules, these data-driven approaches process massive amounts of data to discover underlying patterns to support informed decisions and predictive analytics (Ghahramani, 2015; Jordan and Mitchell, 2015). ML approaches include simple algorithms such as linear regression and more advanced methods such as decision tree (Song and Lu, 2015), SVM (Hearst et al., 1998), and biologically computational models such as artificial neural network (ANN) (Wang, 2003). The design of ANN mirrors the cerebral neural organization into a web of artificial neurons in hierarchical strata. Constructed from multiple layers of interconnected nodes with adjustable weights, ANN replicates brain neural connections into a hierarchical framework that enables ANNs to decode sophisticated relationships hidden in data by stacking abstract representations from raw input signals in successive processing stages (Kos and Krenker, 2011).

Based on these ideas, DL has emerged as a new discipline in ML research. This new discipline aims to use multi-layered ANN to discover sophisticated patterns and abstract representations in data. Compared with conventional shallow architectures, DL models possess deeper architecture and allow for modeling sophisticated functional relationships (Chen, 2015). These models have shown great potential in automated feature extraction, sophisticated representational power and holistic learning framework in processing information from initial input to final output. It is therefore an important research topic in contemporary ML research (Vargas et al., 2018).

The hierarchical architecture of DL model allows autonomous extraction of sophisticated feature representations from raw data. The neural units in successive processing stages transform input signals progressively, and finally reach more abstract patterns and semantic characteristics. This layered transformation process enables AI model to gradually construct a complete representation hierarchy and execute sophisticated computational tasks. Compared with conventional ML models and classical analytical techniques, contemporary advanced models such as convolutional neural networks (CNN) (Lecun et al., 1998), recurrent neural networks (Elman, 1990), generative adversarial networks (Goodfellow et al., 2020), and Transformer (Vaswani et al., 2017) architectures achieve better performance especially when applied to complex problems in visual pattern recognition, acoustic signal interpretation and human language understanding tasks.

## From Literature to Knowledge Graph: Methods and Processes

**Figure 3:**
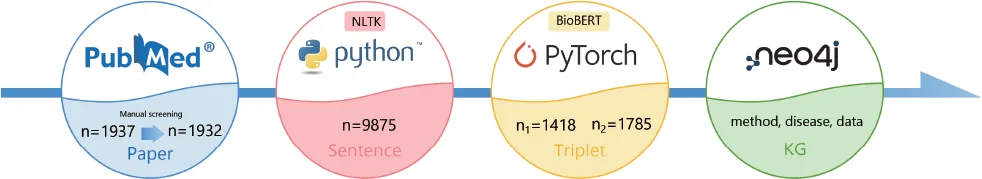
The workflow for constructing the KG from biomedical literature. The process begins with retrieving 1937 articles from PubMed, followed by manual screening to retain 1932 relevant articles. These articles were processed with Python and NLTK, resulting in 9875 segmented sentences. Using BioBERT fine-tuned in PyTorch, entity and relationship extraction generated 1418 triplets in the schema <*method, apply_to, disease*> and 1785 triplets in the schema <*disease, is_related_to, data*>. The extracted triplets were imported into Neo4j for KG construction and visualization. KG: Knowledge graph.

**Figure 3** provides a step-by-step overview of the methodology for constructing the KG from biomedical literature. The workflow highlights the key phases, tools, and intermediate outputs involved in this process.

### Tools and technologies

To achieve our objectives, we utilized the following tools and technologies:

**PubMed:** PubMed (https://pubmed.ncbi.nlm.nih.gov) offers comprehensive access to biomedical literature and was used as our primary source for retrieving studies related to the intersection of AI and mental disorders (Kim et al., 2023b).

**Python:** Python (Python Software Foundation, Wilmington, DE, USA; https://www.python.org) served as the primary programming language for implementing our data processing, model training, and KG-building workflows (Python Software Foundation, 2016). Its robust ecosystem, including libraries like NLTK and PyTorch, made it particularly well-suited for handling natural language and DL tasks in our workflow.

**PyTorch:** PyTorch (Meta Platforms, Menlo Park, CA, USA; https://pytorch.org) was used to build and fine-tune DL models. Specifically, it enabled efficient training of the BioBERT model, taking advantage of GPU acceleration and dynamic computation graphs to optimize named entity recognition (NER) and relation extraction performance (Paszke et al., 2019).

**NLTK (natural language toolkit):** NLTK (NLTK Project, Philadelphia, PA, USA; https://www.nltk.org) provides a wide range of natural language processing features and tools. In our workflow, NLTK was employed for sentence segmentation of combined texts from titles, abstracts, and keywords (Loper and Bird, 2002).

**BioBERT:** BioBERT (Korea University, Seoul, Republic of Korea; https://github.com/dmis-lab/biobert) is a pre-trained biomedical language representation model based on Bidirectional Encoder Representations from Transformers (BERT) (Lee et al., 2020). BioBERT is particularly effective for various biomedical text-mining tasks. In our research, we fine-tuned BioBERT to perform NER and relation extraction from the literature data.

**Neo4j:** Neo4j (Neo4j, Inc., San Mateo, CA, USA; https://neo4j.com) is a highly scalable, native graph database widely used for building KG due to its powerful graph traversal and querying capabilities. We utilized Neo4j to construct and query KG (Miller, 2013).

**Cypher:** Cypher (Neo4j, Inc.; https://neo4j.com/developer/cypher) is a powerful and expressive declarative query language specifically designed for interacting with graph databases like Neo4j (Francis et al., 2018).

### Literature mining

In order to make our research on mental disorders relevant to modern public health and apply an appropriate methodology, we first refer to the Global Burden of Disease studies in 2017–2021 (GBD 2019 Diseases and Injuries Collaborators, 2020) to select high morbidity mental disorders such as depressive disorders, anxiety disorders (AD), SCZ, BD, and attention-deficit/hyperactivity disorder (ADHD). Second, we compared our selection with the diagnostic categories in ICD-11 and DSM-5 classification systems, including but not limited to mood disorders, psychotic disorders, neurodevelopmental disorders and trauma-related disorders. Third, we prioritized disorders for which multimodal data are relatively available, which can support comprehensive and reproducible analysis. In terms of methodological scope, we adopted a broad range of computational and data-driven approaches. These include statistical learning, pattern recognition, expert rule-based systems, and optimization-based methods. At the technical level, both modality-specific and task-oriented approaches such as text mining and image-based methods, are widely used in the studies of psychiatric phenomena. This integrative framework provides robust support for finding clinically relevant patterns and latent structures in mental health data. **Additional Table 1** summarizes the selection criteria for diseases and analytical methods in literature mining.

**Additional Table 1:** Selection criteria for literature mining keyword categories

Category	Source/type	Keyword
Methods	Concept	Artificial intelligence
	Technical paradigm	Machine learning
		Deep learning
		Expert system
		Genetic algorithm
		Reinforcement learning
	Application method	Natural language processing
		Computer vision
Diseases	Concept	Mental disorders
		Psychiatric disorders
	GBD/ICD	Depressive disorders
		Anxiety
		Bipolar disorder
		Schizophrenia
		Autism spectrum disorders
		Attention-deficit/hyperactivity disorder
	DSM-5	Post-traumatic stress disorder
		Obsessive-compulsive disorder
		Sleep disorders

DSM-5: Diagnostic and Statistical Manual of Mental Disorders, Fifth Edition; GBD: Global Burden of Disease; ICD: The International Statistical Classification of Diseases and Related Health Problems.

We use these keywords from **Additional Table 1** to combine the pattern <*method*> AND <*disease*> to construct 8 × 11 groups of keywords. Although there are certain conceptual overlaps between these keywords, they cover commonly used AI methods comprehensively and most representative diseases are included. Experimental articles usually explain clearly what entities and relationships are. While reviewing articles may include multiple studies, its specification and accuracy are not reliable. In order to train entities and relationships extraction from abstracts, we removed review and commentary articles from the retrieval process and kept only experimental and clinical studies. We retrieved the title, abstract and keywords of 1937 relevant articles from PubMed. Subsequently, we manually filtered out articles that did not belong to the research theme or did not have an abstract, and finally, we kept 1932 articles in total related to mental disorders and AI. For each article, we merged its title, abstract and keywords into a single string and split these combined texts into individual sentences using NLTK. Sentence segmentation is the process of splitting a continuous block of text into manageable chunks, thus facilitating more precise and targeted analysis by BioBERT. Finally, we obtained a total of 9875 sentences split from the combined texts of 1932 articles in total. All the sentences were used as individually discrete units of analysis in the subsequent process. To prepare the sentences that could be input into BioBERT, we used a sliding window process. A sliding window process helps to keep the length of the input corpus compatible with the input length limit of the model, and also helps to preserve contextual information near the target sentence. The length of the window was set to 512 tokens to meet the maximum input length of BioBERT, and the stride was set to 256 tokens, i.e. each window overlapped with the previous one by 256 tokens. This design helped maintain contextual continuity between segments. Maintaining contextual continuity between segments is particularly important because it helps to maintain contextual coherence and capture dependencies across the sentence boundary. By using NLTK for text preprocessing and implementing a sliding window strategy, we effectively prepared the textual data for input into BioBERT. These steps ensured that the data was appropriately segmented and contextually enriched, thereby improving the accuracy and effectiveness of the model in downstream tasks.

### Knowledge graph construction

For constructing the KG, we predefined two schemas: <*method, apply_to, disease*> and <*disease, is_related_to, data*>. We used these two schemas as the backbone of our KG. Specifically, we defined the types of entities and relationships that we wanted to extract and represent in our KG. We defined schema <*method, apply_to, disease*> to represent relationships to which we would apply AI methods for different mental disorders. By using ‘*apply_to*’ as the relationship, each triplet can contain the key information about which AI method is applied to which mental disorder and in what context. Therefore, we could systematically extract and understand in what situation AI methods would be applied to different mental health disorders (treatment, research, diagnosis, and rehabilitation). In the second schema <*disease, is_related_to, data*>, we predefined the relationship as ‘*is_related_to*’ due to simple relationships between diseases and datasets. By simplifying this schema, we could achieve higher relationship extraction accuracy.

BioBERT was chosen for NER and relation extraction tasks due to its excellent performance in processing biomedical literature entities and relationships. To better adapt the model for extracting predefined schemas, 300 samples from the data were randomly selected for annotation, and data from the Unified Medical Language System (Bodenreider, 2004) was integrated to enrich the dataset. Unified Medical Language System provides a comprehensive set of biomedical terms and relationships, enhancing the diversity and robustness of the training data. The annotated data, combined with Unified Medical Language System data, was split into three sets: a training set (70%), a validation set (20%), and a test set (10%). The BioBERT model was fine-tuned on the training data for 120 epochs. During training, the cross-entropy loss function was employed to measure prediction errors, and the Adam optimizer was used to update the model parameters, enhancing learning efficiency and accuracy. The performance of the fine-tuned BioBERT model was evaluated using standard metrics for NER tasks. Specifically, the model achieved an accuracy of 90.34%, a recall of 84.53%, and an F1-score of 89.27% on the test dataset. After fine-tuning, a predictor was constructed to perform NER on the entire corpus of literature data. The output was consolidated into triplets, resulting in 1418 <*method, apply_to, disease*> triplets and 1785 <*disease, is_related_to, data*> triplets.

**Figure 4:**
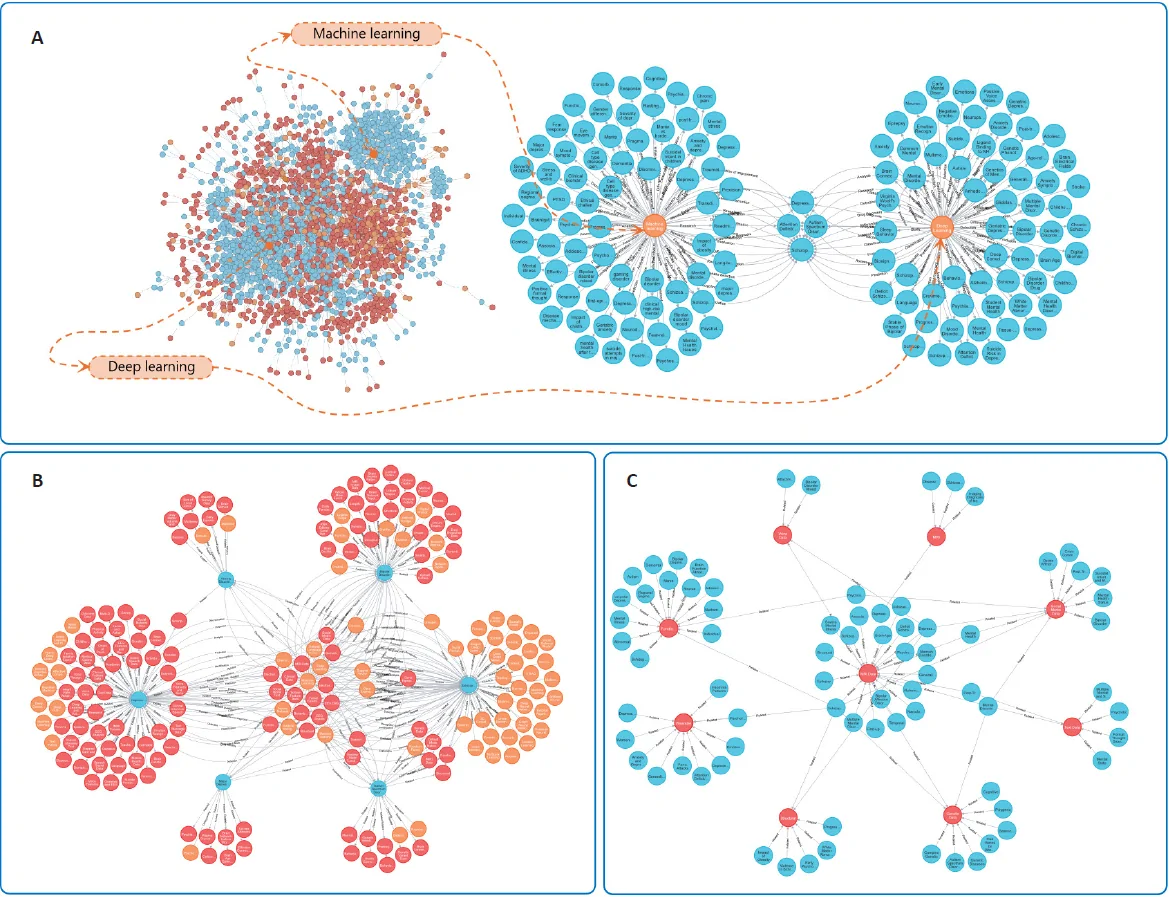
Thumbnail distribution of KG and visualization of relationships of key nodes after retrieval. (A) Distribution of machine learning and deep learning methods with the most associated nodes in the KG. (B) Visualization of the association relationship between five common mental illness nodes. (C) Visualization of data type node association relationships for training models. Using D3.js (v7.0, https://d3js.org/) for interactive and network-based views. KG screenshots were obtained from Neo4j Browser (v4.0, https://neo4j.com/), and the final figure was composed and annotated using Microsoft PowerPoint (v16.0). KG: Knowledge graph.

Neo4j was selected as the graph database system due to its excellent performance and flexibility, which are crucial for the large-scale storage, querying, and visualization of the KG. Its powerful graph traversal and querying capabilities make it an ideal platform for building and interacting with KGs. All extracted entities (methods, diseases, and datasets) were imported into Neo4j as nodes, and relationships between these entities were created using the Cypher query language. By implementing these steps, the KG was effectively constructed and queried, providing a powerful tool for exploring and analyzing the relationships between AI methods and mental health disorders. **Figure 4A** illustrates the thumbnail of the finally constructed KG.

### Visual analysis of knowledge graph

We perform complex queries to analyze relationships within the KG, leveraging the query capabilities of Neo4j. From the overall distribution of nodes in the KG shown in **Figure 4A**, it is evident that the orange nodes (methods) are surrounded by numerous blue nodes (diseases), particularly in the areas labeled ML and DL. This indicates that ML and DL methods are widely applied in various disease studies and are currently prominent methods in this field.

A further network analysis focused on ML and DL reveals that, by examining the specific diseases surrounding these method nodes in **Figure 4B**, ML is associated with diseases such as AD, BD, PTSD, and suicide risk. These diseases are often related to structured data and traditional classification and regression tasks. In contrast, DL is associated with diseases such as ADHD, emotional disorders, and sleep disorders, which frequently rely on complex pattern recognition and the processing of unstructured data. It is also observed that ML and DL are jointly applied in the research of certain diseases. For instance, depression, SCZ, and ASD are investigated using both methods. ML excels at handling structured data, effectively performing classification, regression, and clustering tasks through statistical methods and feature engineering. Meanwhile, DL demonstrates outstanding performance in processing unstructured data (such as images, audio, and text), particularly in pattern recognition and feature extraction. These diseases shown in **Figure 4C** are major research topics in the field of mental disorders, which include highly diverse data types, such as structured data (surveys, clinical tests, etc.), semi-structured data (electronic health records), and unstructured data (text records, images, videos, and audio). The complexity of mental disorders requires capturing various features and patterns in the analysis. Consequently, in practical research, ML and DL methods are often integrated. Studies on mental disorders typically necessitate integrating multiple sources of data. Some researchers combined ML and DL methods to analyze multimodal data, which improved the accuracy of diagnosis and prediction (Amal et al., 2022; Behrad and Saniee Abadeh, 2022). Others constructed hybrid models, DL models were used to extract complex high-level features from data, and ML models were used to implement classification or regression tasks (Chowdhury et al., 2021; Mavaie et al., 2023). Furthermore, cross-disease comparison and comprehensive analysis of the same data type can be performed by combining the two methods to achieve deeper and more intensive research on mental disorders (Sathyanarayanan et al., 2023). By combining ML and DL, researchers can perform more in-depth analysis of the data of mental disorders to better solve the problems of mental disorders.

### Visual analysis of research hotspots

**Figure 5:**
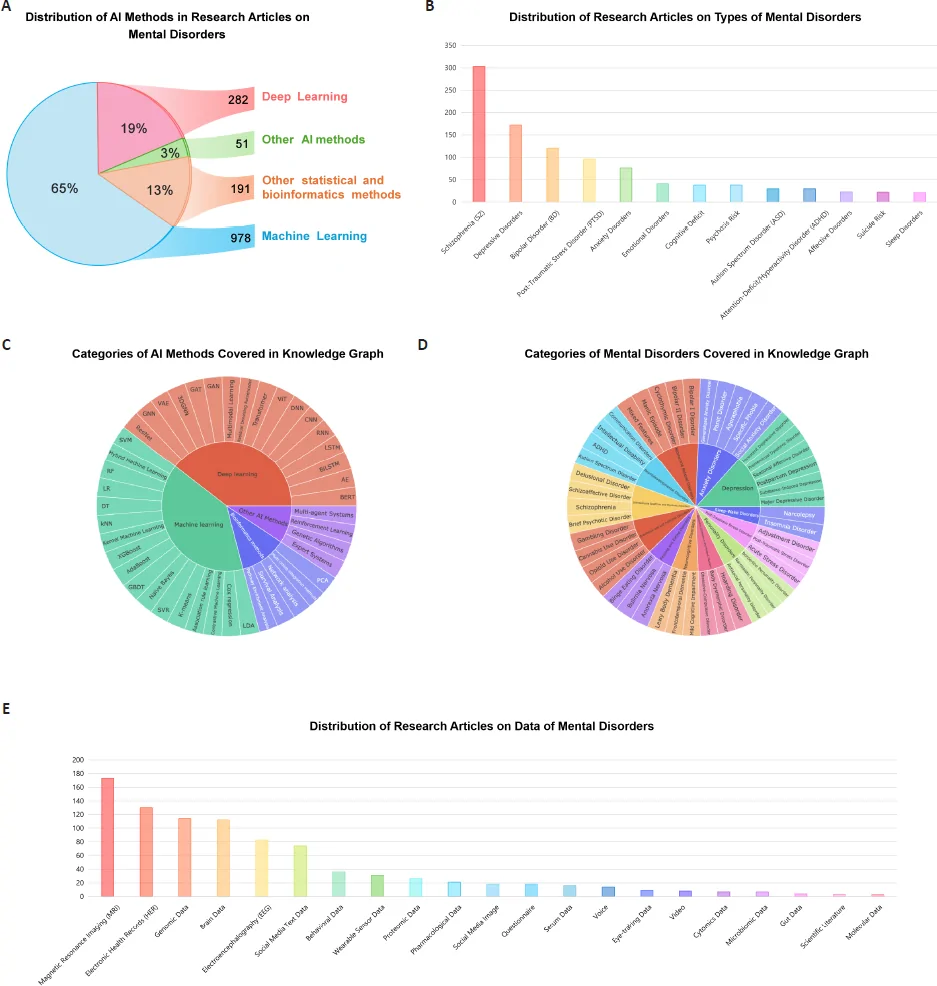
Visualization of classification statistics for methods, diseases, and data entities. (A) Statistics on the frequency of methods used to solve mental disorders in the KG, used to obtain the most commonly used methods. (B) Frequency of studies on mental disorders and disease types, and most popular mental illness studies. (C) For the AI methods included in the KG, the outer circle represents the specific AI method, and the inner circle represents the classification to which the method belongs. (D) In the classification of mental disorders included in the KG, the outer circle represents the specific mental disorders, and the inner circle represents the classification of mental disorders. (E) The most commonly used data forms when training disease-related models of mental disorders. A, B and E were designed and assembled using Microsoft PowerPoint (v16.0). C and D were generated using Python (v3.10) with libraries such as Matplotlib and Seaborn. AI: Artificial intelligence; KG: knowledge graph.

According to KG, we further perform visual analysis on frequently appeared methods, diseases and data in KG as shown in **Figure 5A**. The results show that, compared with diseases, methods have different prevalence. Specifically, ML is the most frequently used method, accounting for 65% of all identified studies. DL also occupies an important position in DL, accounting for 19%. The rest of special AI methods such as evolutionary computation and rule-based system account for 3%. Conventional analytical methods are still valuable when they are intelligently combined with other techniques. These approaches can provide alternative analytical views when used in combination with advanced techniques such as ML and DL. Especially when methods are used in combination with experimental validation in biology, conventional methods still have value when used in combination with experimental validation in biology. Beyond the independent application of ML and DL, in this study, the ML and DL integrated methods are included in the DL category. First, they use a two-stage architecture, in which DL is used to extract features and ML classifier is used for the final classification. Second, they use a multimodal pipeline which combines structured clinical data and unstructured data. For example, long short-term memory (LSTM) is used to extract deep semantic features from text, which are combined with sentiment dictionary features. Depression is classified by logistic regression. The hybrid model achieves better performance than using only one ML or DL model (Ansari et al., 2023). The above phenomena can be explained by the dominance of ML methods from the following perspectives: First, the algorithms of ML are more likely to be robust in few shot datasets. Therefore, they are more likely to be used in structured datasets, such as clinical questionnaires, psychometric scales and behavioral assessments in psychiatric studies. DL models are more likely to be used to handle unstructured or high dimensional data, such as magnetic resonance imaging (MRI) images, electroencephalogram (EEG) records and clinical text. However, DL models need large labeled datasets and many computational resources, which may not be available in all studies. Second, the complexity of DL architectures and the need to rely on dedicated data science and engineering personnel present practical barriers to use, especially in settings lacking dedicated data science or engineering teams. Finally, ML methods are more interpretable, and clinical applications, including psychiatric ones, require that clinical decisions are transparent and explainable. These combined factors likely contribute to the observed distribution of methodological preferences in the field. **Figure 5B** shows that SCZ, depressive disorder, BD, PTSD, and AD are the most studied targets of current AI applications to mental disorders, probably because they are highly prevalent and highly burdensome disorders. In addition, mood disorders, cognitive deficits, psychosis risk, ASD, and ADHD also attract widespread attention. Of course, research on affective disorders, suicide risk, sleep disorders, and other topics is less frequent, but still represents hot spots of interest of many researchers. **Figure 5C** provides a hierarchical classification of the AI-related methods included in the KG constructed. The methods are divided into ML, DL, statistical and bioinformatics techniques, and other approaches. As expected, the methods used in the atlas are highly diverse, and the computational strategies adopted in different contexts in the field of mental health are represented. For instance, SVM, CNN, or logistic regression are frequently used models, but also less common methods such as genetic algorithms, RL, or multi-agent systems are present. **Figure 5D** shows the hierarchical classification of mental disorders represented in the KG. Disorders are divided into major diagnostic groups including mood disorders, AD, psychotic disorders, neurodevelopmental disorders, and others. Under each group, the most common diagnostic subtypes are represented, such as major depressive disorder (MDD), generalized AD, SCZ and ASD. This structure shows the clinical variety of literature and confirms that the KG covers various mental diseases. **Figure 5E** illustrates what types of data are most important in the research intersection of AI and mental disorders. MRI, electronic health records, genomic data, brain data, and EEG are the most used data types. In terms of clinical data types, they are relatively easy to get and many public datasets exist for the genomic data type, which can help researchers to collect data and analyze them. Clinical data usually have high objectivity and authenticity and provide accurate and detailed information to support reliable analysis. By training models with these data types, we can get better results with strong clinical relevance. For instance, we can use MRI data to accurately analyze the changes of brain structure, use electronic health record data to provide comprehensive condition analysis, use genomic data to reveal the genetic basis of diseases, and use EEG data to monitor brain activity. Behavioral data such as social media text, speech, video data, and eye-tracking are also key focuses for researchers. These data types are valuable in mental and psychological health research because they reflect the daily behaviors and emotional states of users. Abnormalities in mental and psychological conditions often lead to behavioral anomalies, and extracting features from these data types using ML or DL methods can capture mental and psychological changes, aiding in diagnosing mental disorders. Proteomics data, pharmacological data, microbiome data, gut data, scientific literature data, and molecular data are applied in specific research fields. These data types usually belong to specific research and they are relatively large and complex. They are hard to collect and can only be obtained by complex experiments. Some data types such as microbiome and gut data, belong to emerging research areas and the research on them started relatively late, so their application frequency is relatively low.

In summary, MRI, electronic health records, genomic data, EEG data, and social media text data are widely used data types in the research of AI and mental disorders. ML and DL methods are particularly preferred by researchers for handling unstructured data and complex pattern recognition tasks. These methods excel in processing these data types and are transforming the intervention approaches for mental disorders such as SCZ, depression, BD, and PTSD, providing new avenues for improving diagnosis and treatment outcomes.

## Harnessing Machine Learning and Deep Learning in the Battle against Mental Disorders

Building on the insights gained from the KG analysis and in conjunction with clinical studies on mental disorders, we filtered the original <*method, apply_to, disease*> triplets according to the following criteria: methods = {machine learning, deep learning} and diseases = {Depression, AD, BD, SCZ, ASD, Sleep Disorders, PTSD, ADHD}. This filtering process resulted in 878 triplets. We then conducted a statistical analysis on the application relationship types, identifying 268 triplets related to early detection (prediction), 225 triplets related to diagnosis (classification, differentiation), 168 triplets related to treatment (management, drug design, patient stratification), 145 triplets related to care, prognosis, and rehabilitation (monitoring, assessment), and 72 triplets related to other aspects (association analysis, exploration, quantification, summarization, etc.). Subsequently, we conducted a comprehensive literature review, summarizing the workflows and specific applications of DL and ML in mental disorders. This analysis is divided into two major fields of application: early detection and diagnosis, and personalized treatment and care. Each part provides a detailed review of the methodologies and research findings in these areas, comparing different approaches and outlining their contributions to the field.

### Workflow of machine learning and deep learning in the field of mental disorders

**Figure 6:**
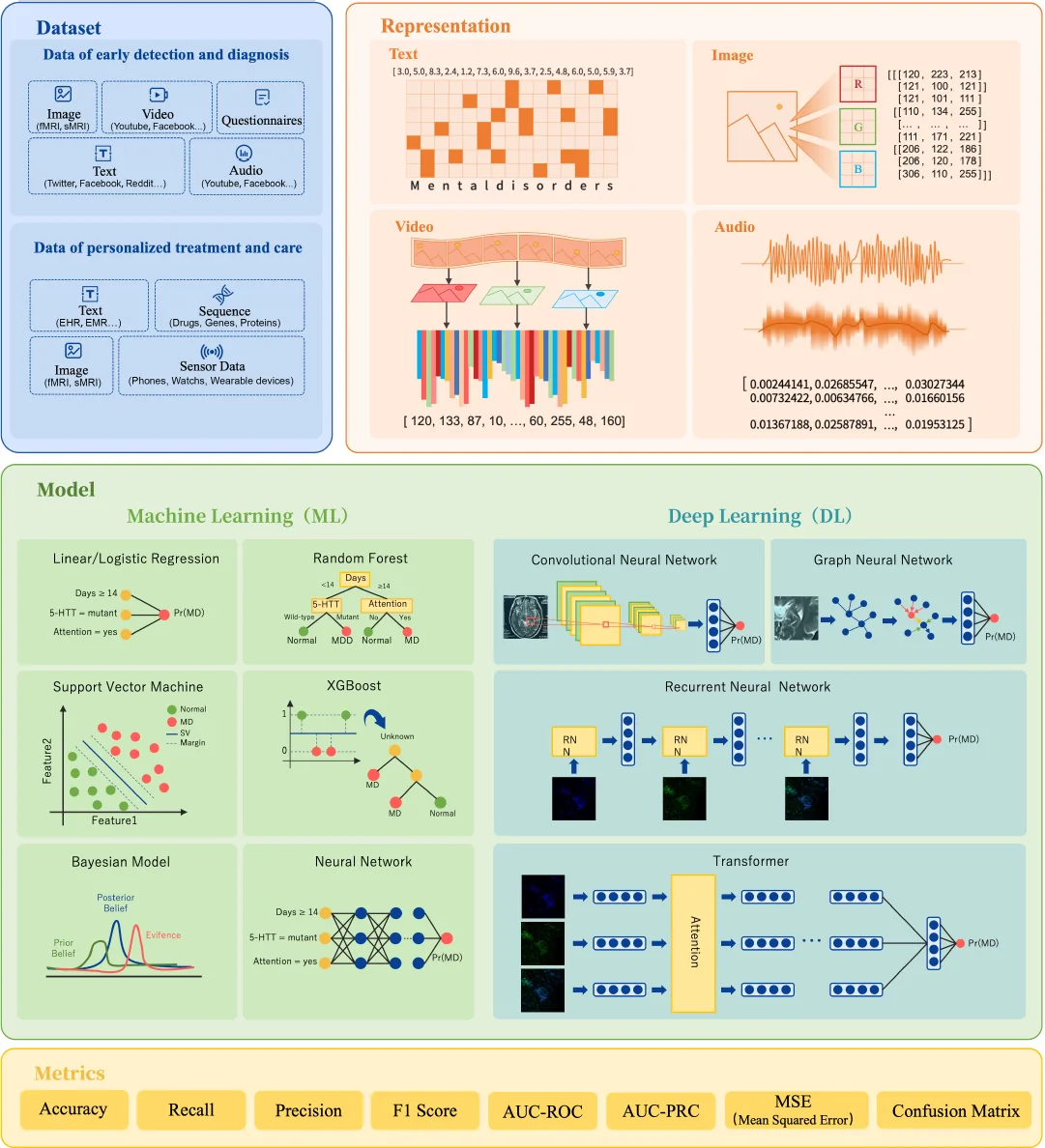
The general architecture of ML and DL application models in mental disorders. The framework is mainly divided into four parts: Dataset, Representation, model and Metric. Dataset: Data sources and formats for different application fields; Representation: Processing the original data source into a vectorized representation that the model can recognize, demonstrating the transformation of text, images, video and audio; Model: Enumerates the commonly used classical machine learning and deep learning methods; Metric: Common indicators for evaluating the performance of models. Created with Microsoft PowerPoint (v16.0). This figure includes icons from the IconPark open-source icon library (https://iconpark.oceanengine.com), used under the terms of the MIT License. AUC-PRC: Area under the precision-recall curve; AUC-ROC: area under the receiver operating characteristic curve; DL: deep learning; EMR: electronic medical record; fMRI: functional magnetic resonance imaging; HER: health electronic records; ML: machine learning; MSE: mean squared error; sMRI: structural magnetic resonance imaging.

**Figure 6** illustrates the general workflow for applying ML or DL in the field of mental disorders, which is broadly divided into steps such as data collection, data representation, model training, model evaluation, and practical application. Initially, clinical text data, imaging data, social media text, audio and video data, genetic data, and wearable device data are collected. Researchers then perform data cleaning operations, such as removing noise, handling missing values, and addressing outliers. Subsequently, these data must be converted into numerical forms suitable for ML or DL algorithms. For instance, text data can be represented as word vectors, image data as RGB channel feature vectors, audio data as time-domain signals or spectral features, and video data as pixel sequences or high-level visual features (Wu et al., 2018; Zhang et al., 2018).

Feature extraction is then performed to prepare the data for subsequent ML or DL models. Features can be manually designed or automatically learned through unsupervised learning or autoencoders, aiming to enable the models to learn comprehensive and useful information (Hasan, 2023; Rawat and Rai, 2023). It is essential to determine whether the specific problem is a regression or classification task and to select appropriate ML or DL models accordingly (Dufourq and Bassett, 2017). For example, linear/logistic regression is used for simple classification and regression tasks such as symptom prediction and risk assessment, while SVM is suitable for complex nonlinear classification problems such as emotion classification (Pochet and Suykens, 2006; Salazar et al., 2012). CNNs are used for handling tasks with grid-structured data, such as MRI image classification, object detection, and semantic segmentation (Pereira et al., 2016). Transformers are suitable for tasks involving long sequence data or multimodal data. Considerations of model complexity and interpretability are crucial (Mallick et al., 2024). Generally, ML is more suitable for the analysis of small-scale, structured data and scenarios requiring high interpretability, while DL excels in handling large-scale, unstructured data, particularly in tasks requiring high precision and complex pattern recognition. Once the model is determined, the dataset is divided into training, validation, and test sets. These partitions are used to train, test, and validate the model, optimizing its parameters and validating its performance. Appropriate metrics are used to evaluate the performance of model, such as accuracy, recall, precision, F1 score, area under the receiver operating characteristic curve (AUC), and area under the precision-recall curve for classification problems, and mean squared error and confusion matrix for regression problems. Based on the evaluation results, the hyperparameters or structure of model may be adjusted. It is important to note that this workflow serves as a general guideline, and specific details may vary depending on the task. Ethical considerations, privacy, data security, and regulatory compliance must also be addressed in practical applications.

### Pioneering early detection and precision diagnosis

Physiological diseases are often mentioned in daily conversations due to their obvious symptoms and public concerns. In contrast, mental disorders belong to psychological illnesses, which usually present with covert symptoms in early stages and usually develop insidiously and gradually (Banner, 2013). Minor emotional fluctuations and behavioral changes in people are usually ignored or mistaken as normal reactions to daily stressors. The symptoms of mental disorders in early stages are usually nonspecific and may also appear in other medical conditions (McGorry et al., 2008). For example, continuous fatigue and poor concentration may be diagnosed as depression but may also be caused by insufficient sleep, anemia, and hypothyroidism (Park and Zarate, 2019). The lack of specific symptoms not only makes it difficult for people to identify their mental disorders but also requires more efforts from clinicians to diagnose mental disorders compared with physical diseases. In addition, diagnosis of mental illnesses usually relies heavily on comprehensive clinical examinations and patients’ self-reports since there are no objective biomarkers for mental disorders and few efficient screening methods at present. Therefore, mental disorders usually cannot be detected by patients themselves or others during initial onset or early stages. Once mental illnesses are detected and diagnosed with a delay, the conditions will become more severe. The increase in psychological stress further aggravates other physical health problems and causes the occurrence or aggravation of somatic comorbidities. Consequently, the early and accurate detection and diagnosis of mental disorders are crucial for effective treatment and recovery.

To address this challenge, researchers have begun to investigate how AI can assist in the early detection and diagnosis of mental disorders in recent years. **Additional Table 2** summarizes representative studies in the last 5 years that applied ML and DL methods in mental disorders research (Chang et al., 2011; Birnbaum et al., 2017, 2020; Jaiswal et al., 2017; Shim et al., 2019; Srinivasagopalan et al., 2019; Yan et al., 2019; Li et al., 2020, 2021; Tadesse et al., 2020; Zhang et al., 2020, 2023a, b; Richter et al., 2021; Wang et al., 2021, 2024; Hayati et al., 2022; Ji et al., 2022; Liu et al., 2022b; Organisciak et al., 2022; Yin et al., 2022; Yoon et al., 2022; Colledani et al., 2023; Rahaman et al., 2023; Han et al., 2024; Helmy et al., 2024; Lee et al., 2024; Malhotra and Jindal, 2024; Xu et al., 2024; Yang et al., 2024; Zhai et al., 2024; Gao et al., 2025). Furthermore, these models have proved their great value not only in reducing the work burden of clinicians but also in discovering subtle patterns that may be unobservable to clinicians during traditional diagnosis. They accept a wide variety of inputs, including both non-clinical inputs (such as images, videos, and texts shared on social media) and clinical inputs (such as scale questionnaires, clinical dialogues, and medical images) (Zeng et al., 2018; Srinivasagopalan et al., 2019; Yan et al., 2019; Li et al., 2020, 2021; Yin et al., 2022).

**Additional Table 2:** Summary of AI in early detection and diagnosis of mental disorders

Target	Data domain	Methodology	Motivation	Reference
SCZ	fMRI	DL: MsRNN	To develop an automated classification method that can improve the accuracy and efficiency of diagnosis	Yan et al., 2019
	fMRI, sMRI	ML: LR, SVM, RF DL: Neural network	To improve the accuracy of machine learning and deep learning in diagnosing schizophrenia	Srinivasagopalan et al., 2019
	Text (Twitter)	ML: Gaussian naïve Bayes, RF, LR SVM	To differentiate Twitter users who disclosed schizophrenia from control users	Birnbaum et al., 2017
	Text	DL: RobIn (DNN with self-attention)	To explore a method for early diagnosis of schizophrenia in order to improve diagnostic accuracy and treatment effectiveness	Organisciak et al., 2022
SCZ	MRI	DL: 3D CNN	To improve diagnostic accuracy in schizophrenia and provide earlier opportunities for intervention	Zhang et al., 2023a
	sMRI, fMRI, SNP	DL: MLP, AE, BiLSTM	To improve classification accuracy of schizophrenia	Rahaman et al., 2023
SCZ, MDD, BD	MRI	DL: MIL, CNN	To achieve early detection of serious mental illness, thereby helping to prevent the progression of the disease and reduce the risk of long-term disability	Zhang et al., 2023b
BPD	MRI	ML: SVM	A binary classification task: to develop a reliable and accurate diagnostic tool for BPD that can be used in clinical settings	Li et al., 2020
FEP, BD	sMRI	DL: CNN	To develop an automatic diagnosis method for classifying FEP, BD and health control using sMRI	Li et al., 2021
ADHD	fMRI	ML: XGBoost, multilayer community detection, network analysis	To identify alterations in dynamic brain reconfiguration in children with ADHD and to differentiate subjects with ADHD from typically developing children and to predict ADHD severity of individuals	Yin et al., 2022
ADHD, ASD	Video	ML: SVM DL: Dynamic deep learning	To provide a fully automatic end-to-end system for directly predicting ADHD and ASD in adults	Jaiswal et al., 2017
SSD, MD	Text and image (Facebook)	ML: LR, RF	To identify signals related to mental illness by analyzing text and image data on Facebook, and to provide opportunities for early intervention	Birnbaum et al., 2020
Depression	Text (Twitter)	ML: LGB, RF, SVM, LR	Detection of depression through machine learning methods for early detection and intervention	Helmy et al., 2024
	Text	DL: BERT, LSTM	For early depression detection in social media streams	Wang et al., 2024
	Text	DL: GPT-3	To construct a depression detection model for Malay dialects based on GPT-3	Hayati et al., 2022
	Video and Audio (YouTube)	DL: Transformer, Cross-attention mechanism	To detect depression from non-verbal behaviors	Yoon et al., 2022
	Audio	Expert system	To explore the feasibility of using voice analysis technology for non-invasive early diagnosis of mental illness	Chang et al., 2011
SI, depression	Text (Reddit)	DL: BERT, CNN, RNN, self-attention	To facilitate the automatic detection of mental disorders in online social content	Ji et al., 2022
	Text (Twitter, Reddit)	DL: Transformer	To provide interpretability to enhance the application of the Transformer model in the detection of depression and suicidal behavior	Malhotra and Jindal, 2024
AD, depression	Clinical data, Questionnaires	ML: RF	To provide an objective mechanistic diagnostic tool to differentiate clinical anxiety and depression	Richter et al., 2021
BD, depression	Video, voice and text	DL: multi-DDAE	To help detect symptoms early, provide biomarkers for diagnosis, and assist psychologists in diagnosing during face-to-face interviews	Zhang et al., 2020
MDD	Image	ML: HYDRA (based on SVM)	To identify and validate neuroanatomical subtypes of MDD	Gao et al., 2025
	Clinical data, Questionnaires	ML: DT	To predict whether a patient is suffering from depression	Colledani et al., 2023
	sMRI	DL: 3D-DenseNet	To provide an accurate and rapid method for diagnosing major depressive disorder	Wang et al., 2021
	MRI	DL: DNN	To identify MDD	Liu et al., 2022b
MDD, PTSD	EEG	ML: SVG	To classify PTSD and MDD	Shim et al., 2019
MDD, BD	sMRI and DNA	ML: XGBoost, LightGBM	To identify and diagnose MDD and BD more accurately	Lee et al., 2024
	WES	DL: Vision Transformer, Inception-V3, ResNet50		
Mental disorders	Text (Weibo, Sina Weibo)	DL: Chinese MentalBERT	To propose a model dedicated to the analysis of mental health texts in Chinese to aid early detection of mental health problems and provide interventions	Zhai et al., 2024
	Text	DL: MentaLLaMA	To predict mental health conditions and generate detailed explanations	Yang et al., 2024
SI	Text (Reddit)	ML: Gaussian naïve Bayes, SVM DL: LSTM, CNN, LSTM-CNN	To build an effective model for a suicide risk assessment in various text classification tasks	Tadesse et al., 2020
	Text (Reddit)	DL: Mental-Alpaca	To achieve prediction from the simplest binary classification (presence or absence of psychological risk) to a more complex five-level suicide risk rating	Xu et al., 2024
CD	EEG and multi- omics data	ML: SVM	For personalized diagnosis for neurocognitive disorders	Han et al., 2024

3D: Three-dimensional; AD: anxiety disorder; ADHD: attention-deficit/hyperactivity disorder; AE: autoencoder; AI: artificial intelligence; ASD: autism spectrum disorder; BD: bipolar disorder; BERT: bidirectional encoder representations from transformers; BiLSTM: Bidirectional long short-term memory; BPD: borderline personality disorder; CD: cognitive disorders; CNN: convolutional neural network; DDAE: deep denoising autoencoder; DL: deep learning; DNN: deep neural network; DT: decision tree; EEG: electroencephalography; EHR: electronic health record; FEP: first-episode psychosis; fMRI: functional magnetic resonance imaging; HYDRA: heterogeneity through discriminative analysis; LGB: light gradient boosting machine; LR: linear regression; LSTM: long short-term memory; MD: mood disorders; MDD: major depressive disorder; MIL: multi-instance learning; ML: machine learning; MLP: multilayer perceptron; MRI: magnetic resonance imaging; MsRNN: multi-scale recurrent neural network; PTSD: post-traumatic stress disorder; RF: random forest; RNN: recurrent neural network; SCZ: schizophrenia; SI: suicide ideation; sMRI: structural magnetic resonance imaging; SNP: single nucleotide polymorphism; SSD: schizophrenia spectrum disorders; SVM: support vector machine; TDC: typically developing children; WES: whole exon sequencing.

Non-clinical data often encompass extensive behavioral information that can reflect a wide range of potential abnormal behaviors and emotional changes. For instance, a 2020 study utilized language and image data from Facebook to identify signs of mental disorders (Birnbaum et al., 2020). ML algorithms processed 3,404,959 Facebook messages and 142,390 images from 223 participants, including individuals with SCZ spectrum disorders or mood disorders, as well as healthy volunteers. Using ML algorithms, the study was able to distinguish diagnostic categories based solely on Facebook activity, predicting outcomes more than a year before the first hospitalization, achieving an AUC ranging from 0.72 to 0.77. Birnbaum et al. (2017) analyzed text data from the Twitter timelines of 671 users who self-disclosed diagnoses of SCZ. They developed classifiers using various ML techniques and benchmarked their performance against expert assessments. Among these, the RF classifier achieved the highest performance with an average AUC of 0.88. Similarly, proposed a DL approach that integrated LSTM networks with CNNs, enhanced by word2vec embeddings, to detect suicidal ideation in Reddit posts. This model achieved an accuracy of 0.938. Zhang et al. (2020) introduced a multimodal DL framework that incorporates facial expressions, gestures, audio cues, and linguistic data to support early diagnosis of mental disorders. By leveraging cross-modal correlations, the system achieved an accuracy of 0.893 in depression detection tasks. Notably, the use of non-clinical data provides a unique opportunity to investigate how AI models can detect and respond to mental health symptoms across diverse cultural contexts. As cultural norms strongly influence how emotional distress is expressed in language, behavior, and social media activity, several studies have begun to incorporate culturally specific datasets into AI models. For instance, Helmy et al. (2024) proposed five ML models such as RF and SVM to detect depression in English and Arabic Twitter texts. The experimental results show that the best precision and AUC are 0.983 and 0.996, respectively. The method has high accuracy in depression detection, providing a potential pathway for early intervention and support. Hayati et al. (2022) explored depression detection on dialectal Malay speech using GPT-3. The authors collected and annotated clinical interview data across three regional dialects in Malaysia, GPT-3 achieved the highest accuracy of 0.73 on the combined dataset using four few-shot examples, their findings revealed that model performance varied significantly across dialects. It can be seen that the inclusion of cultural and linguistic diversity in the design of AI for mental health applications is also a factor to be considered. In addition to behavioral signals, neuroimaging-derived non-clinical data have demonstrated considerable potential in characterizing subtypes of mental disorders and optimizing individualized treatment strategies. Gao et al. (2025) proposed a semi-supervised ML approach named heterogeneity through discriminative analysis (HYDRA), which identified two neuroanatomical subtypes of MDD based on fractional anisotropy metrics derived from diffusion tensor imaging. The stability of the subtype classification was supported by an adjusted Rand index of 0.62. Further analysis revealed that incorporating subtype information significantly improved the performance of treatment response prediction models, outperforming those trained on the overall MDD population. Furthermore, LLMs known for their impressive performance in various fields have been applied to mental health. Ji et al. (2022) explored this by training and releasing two pre-trained masked language models, MentalBERT and MentalRoBERTa, based on mental health-related corpora from Reddit, Twitter, and MSM, improving classification performance in the mental illness domain. The MentalBERT and MentalRoBERTa models achieved accuracy rates of 94.58% and 94.33%, respectively, in depression detection. Zhai et al. (2024) proposed a pre-trained language model named Chinese MentalBERT, which underwent domain-adaptive pre-training on the Weibo dataset, a Chinese social media platform, to enhance its efficiency. The model achieved an accuracy of 85.71% in the suicide risk classification task. Yang et al. (2024) introduced MentaLLaMA and constructed the Interpretable Mental Health Instruction (IMHI) dataset with 105K annotated posts and ChatGPT-generated rationales to predict mental health conditions and generating detailed explanations. MentaLLaMA-chat-13B approaches or surpasses SOTA discriminative models like MentalRoBERTa in prediction correctness. This work marks a significant step toward transparent, generalizable, and low-cost LLM-based mental health support systems. Xu et al. (2024) introduced Mental-Alpaca, a multi-task fine-tuned model that is 16.7% higher than GPT-3.5 in terms of balance accuracy, when conducting a comprehensive evaluation of mental health predictions for LLMs. Their findings highlight the effectiveness of multi-dataset fine-tuning and provide actionable guidelines for applying LLMs in the mental health domain. Malhotra and Jindal (2024) introduced a Transformer-based explainable AI model aimed at understanding and interpreting the behavior of depressed and suicidal users on online social networks. This model achieved an impressive accuracy of 0.967 on the test dataset. Wang et al. (2024) developed a model named ESDM, which combines DL techniques of BERT and LSTM with an attention mechanism, for early depression risk detection in social media data streams. The ESDM model demonstrated significant improvements in both early detection and accuracy.

Non-clinical data are often used primarily for behavior analysis and early identification of mental disorders. In contrast, clinical data have accurate medical information and diagnostic outcomes. Therefore, clinical data are more reliable, more structured and more suitable for medical diagnosis applications. Richter et al. (2021) introduced an ML-based psychiatric diagnostic tool using the RF algorithm to analyze clinical questionnaire data, distinguishing between patients with anxiety and depression. The classification success rate of the anxiety group reached 0.805, and that of the depression group reached 0.665. To assist clinicians in better understanding the diagnostic process, Organisciak et al. (2022) developed a deep neural networks model with an attention mechanism based on DSM-5 criteria. Their model achieved an AUC score of 0.983, which was significantly higher than traditional baseline ML methods, indicating a strong possibility of its application as an auxiliary diagnostic tool in clinical practice. Colledani et al. (2023) used the widely-used Patient Health Questionnaire-9 to train and evaluate a decision tree classifier targeted at predicting the diagnosis of MDD. Their model achieved an accuracy of 75% and an AUC of 0.71 on the evaluated dataset. Zhang et al. (2023b) used a DL framework called Multiple Instance Learning, employing MRI data to distinguish patients with severe mental illness from healthy individuals. Validated across two independent datasets, their model demonstrated an AUC of 0.82, the author further explained that the brain regions most focused on by the model when learning MRI features are the default mode network, somatomotor network, and regions related to visual and emotional integration. In another study by Zhang et al. (2023a), a three-dimensional convolutional neural network named SE-VGG-11BN was used for SCZ diagnosis using T1-weighted MRI scans, achieving a remarkable AUC of 0.987. At the same time, in the interpretability analysis, the image areas that the model focuses on in the prediction process are further visualized, mainly including hippocampus, amygdala, thalamus and striatum. Wang et al. (2021) extended the two-dimensional DenseNet to a three-dimensional DenseNet to better employ the three-dimensional whole-brain structural information of structural MRI data for the diagnosis and analysis of MDD, achieving an AUC of 0.86. Ma et al. (2024) applied an ML method called CatBoost to analyze a large set of metabolomics data for classifying depression patients. When ranking the importance of classification features, they found that the most significant top 20 proteins were mainly concentrated in the cytokine-cytokine receptor interaction pathway, and this model achieved an AUC of 0.716. Unlike those studies that study the interpretability of models by identifying the importance of the hippocampus, corpus callosum, amygdala and key neural circuits, another research route is to adopt feature engineering methods to preprocess MRI and fMRI data so that the model can better locate brain regions related to disease. Liu et al. (2022b) demonstrated that it is feasible to classify MDD using a deep neural network model based on structural and functional MRI data. Before modeling, they conducted a meta-analysis to screen out key regions of interest to replace whole brain feature input, and particularly included the pons in the model. Finally, the model based on structural volume data of the left inferior frontal gyrus, right amygdala, and pons achieved a classification accuracy of 72.8%, which is the first study to prove that pons play a high importance in MDD classification. Shim et al. (2019) developed an SVM model for distinguishing PTSD from MDD. Its input features are derived from the P300 index extracted by EEG and the current source density of cortical source localization. Source localization analysis focuses on cortical areas such as anterior cingulate gyrus, middle frontal lobe, parietal precuneus and lingual gyrus. The characteristics of these cortices not only improve the overall accuracy, but also significantly enhance the model’s sensitivity for PTSD patients, with an accuracy rate of over 80%.

Although single-modal models perform well, they still have limitations in capturing the multifaceted nature of mental disorders. Therefore, recent research has begun to turn to multi-modal integration, combining neuroimaging, genomic and other biological data to improve diagnostic accuracy and deeper understanding of disease mechanisms. Rahaman et al. (2023) improved SCZ diagnosis accuracy by integrating multi-modal data including structural MRI, functional MRI, and genomic data using autoencoders combined with bidirectional LSTM networks, reaching a diagnostic accuracy of 92%. The authors also conducted interpretable interpretations identifying the hippocampus, corpus callosum, amygdala, and subcortical-cortical circuits as key contributors to the classification of SCZ. Han et al. (2024) constructed a comprehensive diagnostic model based on the SVM algorithm by combining multi-modal EEG data with metagenomics, proteomics, and metabolome data from serum and hippocampal tissue. This multi-modal model achieved an accuracy of 92.69% and an AUC of 0.9641. Lee et al. (2024) developed a multi-modal fusion model using Transformers and ML algorithms to process neuroimaging and genomic data respectively, for the diagnosis of MDD and BD. The performance of the multi-modal fusion model surpassed that of single-modal models, with an AUC of 0.8904.

There is no doubt that ML and DL methods can discover and diagnose mental disorders more quickly and efficiently than traditional clinical assessments. By analyzing non-clinical data generated from online social platforms, such as text, audio, and video, these methods can extract behavioral patterns, emotional changes, and social media activity, providing rapid and accurate judgments in the early identification of mental disorders. This enables timely interventions, gaining valuable time for early treatment. Additionally, by analyzing clinical data such as medical imaging, genetic information, and multi-omics data, these methods can predict and classify various mental disorders, assisting clinicians in making faster and more accurate diagnoses. In particular, DL methods excel at extracting complex information from data, such as images and videos from social platforms as well as medical imaging. The integration of non-clinical and clinical multimodal data offers richer insights and enhances processing accuracy compared to single-modal approaches (Heiliger et al., 2022).

However, current research largely focuses on the multimodal fusion of non-clinical or clinical data separately, with relatively limited attention given to the fusion of multimodal data across both clinical and non-clinical domains. Effectively integrating non-clinical and clinical data to provide more comprehensive and precise diagnoses remains an area worthy of further exploration. To advance this goal, future work could explore fusion strategies such as early fusion with shared encoders, late fusion through ensemble learning, and cross-modal attention mechanisms for semantic alignment between modalities (Xue and Zhu, 2024; Zhao et al., 2024b). Heterogeneous graph neural networks may also offer a promising approach to structurally model relationships across diverse data sources, such as symptoms, behaviors, and clinical indicators (Zhang et al., 2019; Shao et al., 2023).

### Personalized treatment and intervention strategies

Specific medications or psychotherapies may be effective for one subgroup of patients but ineffective for another subgroup with the same diagnosis. In the evolving era of evidence-based psychiatry tailored to individual patients, In the evolving era of evidence-based psychiatry tailored to individual patients, ML and DL algorithms can also uncover the complex pathophysiological mechanism of mental disorders by analyzing objective, measurable intrinsic phenotype data. These advancements facilitate personalized treatment options, virtual psychotherapy, medication support, and the adjustment of intervention plans, ultimately helping to alleviate the burden of disease (Ausman, 2019; Lee et al., 2021). ML and DL offer robust tools for clinical prediction at the individual level. A notable success is the use of the gradient boosting decision tree algorithm by Rajpurkar to construct an ML model that predicts the efficacy of antidepressant treatment using EEG and Hamilton Depression Rating Scale scores (Rajpurkar et al., 2020). Additionally, web-based cognitive-behavioral therapeutic apps have become a widely used treatment method. Furthermore, more virtual and robotic AI agents are being developed for high-level therapeutic interventions. For example, Ewbank et al. (2021) employed a DL model to automatically encode session transcripts from Internet-enabled cognitive behavioral therapy for approximately 34,000 patients. The study aimed to explore the relationship between patient language and therapeutic outcomes, achieving an optimal F1 score of 0.940. Thieme et al. (2023) developed a recurrent neural network-based app to predict treatment outcomes for patients undergoing internet-provided cognitive behavioral therapy for depression and anxiety symptoms. Researchers have introduced the term M2Health to describe mobile health systems specifically designed for mental health. M2Health can potentially enhance formal clinical evaluations of mental disorders by providing clinicians with summaries of patient remote monitoring technology data collected shortly before appointments (Han et al., 2021). Kim et al. (2023a) developed a smartphone application that collects digital biomarker data reflecting the social and behavioral activities of adolescents. They used a deep neural network to analyze the collected data to predict treatment responses for adolescent depression, achieving a testing accuracy of 77% on an unseen dataset. ML and DL are more mature and widely used in drug response prediction, and to some extent help clinicians solve the problem of “different patients respond to drugs differently under the same diagnosis.” These models employ a wide range of data modalities and analytical perspectives to predict individual responses to pharmacological treatments. Liu et al. (2022a) designed a framework combining graph theory analysis with SVM to predict treatment response to antipsychotic drugs in patients with first-episode SCZ. Using resting state functional MRI data, they quantified whole-brain degree centrality, focusing on key regions and the striatum-frontal circuitry. This framework achieved 84.2% accuracy and an AUC of 0.925 when distinguishing treatment-effective and ineffective patient populations. Using ML and multi-omics methods, Joyce et al. (2021) predicted the response of patients with MDD to combined antidepressant therapy, achieving an accuracy of 76.6% and an AUC of 0.84 using the XGBoost model without single nucleotide polymorphisms. Including single nucleotide polymorphisms increased the accuracy to 77.5%, and the authors further revealed that sphingomyelin may affect antidepressant efficacy by regulating neurotransmitter vesicles, membrane structure, or synaptic function. Cearns et al. (2022) used linear regression and RF to predict the response of SCZ and MDD patients to lithium treatment, with the models achieving AUC values of 0.5895 and 0.6365, respectively. Ciprian et al. (2020) employed ML methods such as SVM, RF, and linear discriminant analysis to extract features from pre-treatment EEG data to predict the response of chronic treatment-resistant SCZ patients to clozapine therapy, with SVM achieving the highest accuracy at 93.75%. Bao et al. (2021) used various ML methods, including SVM, RF, KNN, and decision tree, to predict the efficacy of repeated intravenous ketamine in MDD patients, with SVM achieving an AUC of 0.86. Ambrosen et al. (2025) used multiple linear regression models to explore the relationship between extracting facial action units from videos, clustering, and positive, negative, and general symptoms of schizophrenia and their response to treatment. The correlation coefficient was 0.77. Tsai et al. (2022) employed a deep neural network model to predict the treatment efficacy of antidepressants in drug-naive and first-diagnosed patients with MDD. The model was trained and evaluated using signature features from various domains, including clinical characteristics, peripheral biochemistry, psychosocial factors, and genetic polymorphisms. The best-performing model achieved an AUC of 0.825. In addition to predicting drug treatment response, ML and DL methods have been explored for predicting the effectiveness of neuromodulation therapies, multi-modal data integration, hospital-based outcome prediction, wearable-based monitoring, and even brain-computer interface applications, as well as pathogenesis mining and therapeutic target discovery. Zandvakili et al. (2019) used SVM to analyze cortical EEG data from the frontal, parietal, and occipital lobes recorded before and after transcranial magnetic stimulation treatment to predict clinical responses to transcranial magnetic stimulation in patients with comorbid PTSD and MDD, achieving classification accuracy exceeding 73% across different frequency bands. Kong et al. (2021) trained a temporal graph CNN using clinical and functional MRI data from patients that capture discriminative topological and temporal features of brain networks to predict treatment response in MDD, the model achieved an accuracy of 89.63% and an AUC of 0.923. Further analysis revealed that the hippocampus and amygdala were key regions influencing classification performance. ML and DL have applications ranging from hospital wards to home wearable devices. Morel et al. (2020) developed an XGBoost-based model to predict readmission in patients with mental or substance use disorders, achieving an AUC of 0.737. Koutsouleris et al. (2021) proposed a multi-modal ML approach integrating clinical, neurocognitive and structural MRI, data to predict SCZ episodes in patients with recent-onset depression. The model identified neurobiological markers such as hippocampal volume reduction, gray matter alterations in the corpus callosum and fronto-temporo-parietal regions, and amygdala abnormalities related to emotional processing, which may predict psychosis transition. The accuracy rate of this method reached 80% and AUC results were as high as 90%. Mullick et al. (2022) proposed a predictive model using various ML algorithms to analyze passive sensor data from mobile wearables and Patient Health Questionnaire-9 depression scores, achieving a maximum accuracy of 89% in predicting changes in depression symptoms and a mean absolute percentage error of 0.27. Coutts et al. (2020) investigated methods for predicting psychological and general health status using heart rate variability data from wearables, proposing a DL model based on LSTM networks, which achieved a classification accuracy of 83% and an AUC of 0.85. Rashmi and Shantala (2023) introduced a brain-computer interface application named Brain-Computer Interface-Assistive Mental Stress Healing (BCI-AMSH), using ML and DL to detect and classify EEG signals and treat psychological stress, achieving a therapeutic efficacy of 76.4%.

Overall, ML and DL are increasingly integrated with a broad range of technologies and devices to collect diverse data from patients during the treatment, intervention stages of mental disorders. These tools effectively analyze multimodal data in psychiatry. Unlike the early detection and diagnosis process, these models focus more on changes in data, such as variations in drug response, EEG changes before and after treatment, changes in brain region and sensor metric fluctuations. ML and DL can be extensively and comprehensively applied to personalized treatment and intervention (**Additional Table 3**; Zandvakili et al., 2019; Ciprian et al., 2020; Coutts et al., 2020; Morel et al., 2020; Rajpurkar et al., 2020; Bao et al., 2021; Ewbank et al., 2021; Joyce et al., 2021; Kong et al., 2021; Koutsouleris et al., 2021; Cearns et al., 2022; Liu et al., 2022a; Mullick et al., 2022; Tsai et al., 2022; Kim et al., 2023a; Rashmi and Shantala, 2023; Sharma et al., 2023; Thieme et al., 2023; Xu et al., 2023; Ajith et al., 2024; Ambrosen et al., 2025). By analyzing brain structure, physiological sensors, facial expressions, voice, smartphone data, and wearable biosensors, these technologies can monitor real-time conditions and support clinical decision-making. This contributes to the development of accurate, objective, and personalized patient risk models, improving triage outcomes, providing comprehensive treatment plans, and precisely predicting drug responses. Such advancements benefit precision medicine and evidence-based medicine.

**Additional Table 3:** Summary of AI in personalized treatment and care for mental disorders

Target	Data domain	Methodology	Motivation	Reference
Depression	Pre-treatment symptom score and EEG	ML: GBDT	To explore the potential of machine learning methods to predict the improvement of individual symptoms by antidepressants	Rajpurkar et al., 2020
	Smartphone sensor data	DL: DNN	To provide a non-invasive method to predict diagnosis and treatment response in depression	Kim et al., 2023a
	Mobile phone sensor data, wearable device data	ML: LR, RF, DT, AdaBoost, XGBoost	To predict changes in adolescent depression scores and depression levels	Mullick et al., 2022
	EHR	ML: L2 norm regularized logistic regression, Naive Bayes, RF, GBDT	To accelerate personalized medical management for patients with mental illness	Xu et al., 2023
MDD	Genomic data	ML: XGBoost	To identify key predictors of antidepressant response	Joyce et al., 2021
	WGS	ML: SVM, RF, kNN, LR, DT	To predict response to ketamine treatment in patients with MDD	Bao et al., 2021
	Clinical characteristics and biochemical indicators	DL: MLP	To predict the therapeutic effects of antidepressants	Tsai et al., 2022
	fMRI	DL: GCN	To explore the application of neuroimaging biomarkers in the prediction of treatment response in MDD	Kong et al., 2021
AD, depression	EHR	DL: RNN	To predict the likelihood of a patient experiencing a significant reduction in mental health symptoms during treatment	Thieme et al., 2023
	Text	DL: BiLSTM	To analyze a large number of conversation recordings and discover potential factors associated with clinical outcomes	Ewbank et al., 2021
Stress, AD, depression	Sensor data	DL: LSTM	To explore the feasibility of using HRV data on wearable devices to predict mental health problems	Coutts et al., 2020
Stress, AD	EEG	ML: SVM, LDA, DT, kNN, Naive Bayes DL: LSTM, CNN	To develop a brain-computer interface-based assistive application for the treatment of psychological stress and anxiety	Rashmi and Shantala, 2023
PTSD, MDD	EEG	ML: Lasso regression, SVM	To predict individual response to repetitive transcranial magnetic stimulation treatment	Zandvakili et al., 2019
SCZ	fMRI	ML: SVM	To predict treatment outcomes for SCZ patients receiving antipsychotic medication treatment	Liu et al., 2022a
	sMRI, PRS	ML: NeuroMiner (an ML integration toolbox)	Predicting the probability of SCZ episodes in patients with clinically high-risk status and patients with recent-onset depression	Koutsouleris et al., 2021
	EEG	ML: SVM, RF, LDA	To provide a non-invasive method to predict patient response to treatment, thereby helping physicians choose treatment options more accurately	Ciprian et al., 2020
	Video (AUs)	ML: LR	To evaluate the symptoms of patients with schizophrenia and predict treatment response	Ambrosen et al., 2025
BD	Clinical data and genotype data	ML: LR, RF	To explore the application of multimodal data and machine learning to predict the response of patients with bipolar disorder to lithium salt therapy	Cearns et al., 2022
M/SUD	Text (diagnostic record)	ML: XGBoost	To predict the risk of hospital readmission in patients with mental or substance use disorders	Morel et al., 2020
ASD, ADHD, ID	Sensor data	ML: DT, SVM, RF DL: CNN, RNN	To analyze sensor data to provide valuable insights into understanding and responding to the challenges faced by people with ASD, ADHD, and ID	Sharma et al., 2023
Mental health	fMRI	DL: CNN	To assess and measure mental health, guide individualized interventions and monitor treatment response	Ajith et al., 2024

AD: Anxiety disorder; ADHD: attention-deficit/hyperactivity disorder; AI: artificial intelligence; ASD: autism spectrum disorder; AU: action unit; BD: bipolar disorder; CNN: convolutional neural network; DL: deep learning; DNN: deep neural network; DT: decision tree; EEG: electroencephalography; EHR: electronic health record; fMRI: functional magnetic resonance imaging; GBDT: gradient boosting decision tree; GCN: graph convolutional neural network; HRV: heart rate variability; ID: intellectual disability; kNN: K-nearest neighbor; LDA: linear discriminant analysis; LR: linear regression; LSTM: long short-term memory; M/SUD: mental or substance use disorder; MDD: major depressive disorder; ML: machine learning; MLP: multilayer perceptron; NLP: natural language processing; PRS: polygenic risk score; PTSD: post-traumatic stress disorder; RF: random forest; RNN: recurrent neural network; SCZ: schizophrenia; sMRI: structural magnetic resonance imaging; SVM: support vector machine; SVR: support vector regression; WGS: whole genome sequencing.

## Artificial Intelligence–Driven Clinical Trials in Mental Disorders: Clinical Research Analysis

Currently, research literature on the optimization of AI algorithms, feature engineering, and the development of ML and DL models in mental disorders has significantly contributed to advancing AI technology from the laboratory to clinical applications (Squires et al., 2023). To further investigate the clinical progress of AI in the treatment of mental disorders, we collected data from ClinicalTrials.gov, the world’s largest clinical trial registry platform (Zarin et al., 2011). This dataset includes 518 relevant studies on AI in mental disorders, comprising 306 interventional studies and 212 observational studies. Interventional studies refer to those where AI-assisted treatments are actively implemented, such as using AI technologies to recommend treatment plans or optimize medication choices, with the aim of verifying the effectiveness of AI in actual clinical settings (Chalasani et al., 2023; Igwama et al., 2024). These studies typically adopt randomized controlled trial designs to assess the impact and effectiveness of AI-based treatments for patients with mental disorders. Observational studies, on the other hand, do not involve any treatment or intervention. Instead, they analyze clinical data and patient information (e.g., medical imaging, social media dynamics, facial information) using AI techniques for the diagnosis and prediction of mental disorders (Lee et al., 2021). The goal of these studies is to identify potential risk factors, disease progression, and the supportive role of AI in clinical treatment through the observation and analysis of naturally occurring data. Data was processed using libraries such as Matplotlib, Numpy, and Plotly for segmentation, and Fuzzywuzzy was used for semantic classification and matching of fields, with results visualized accordingly.

**Figure 7:**
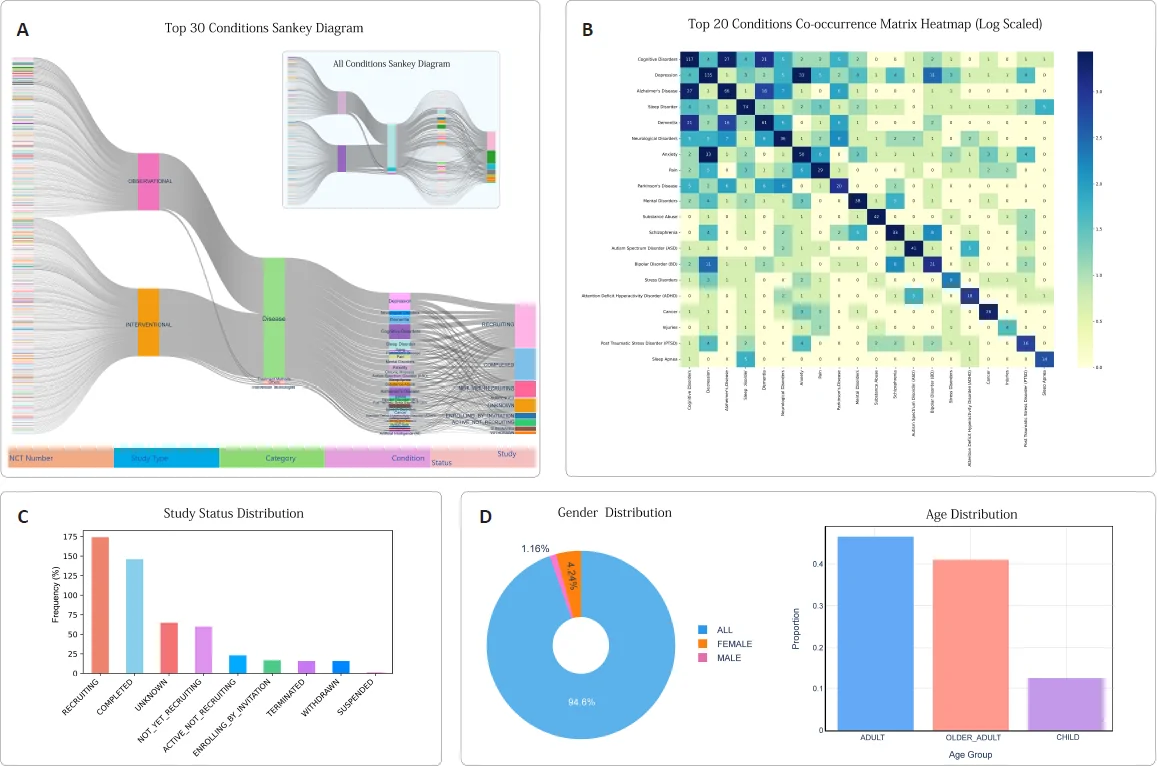
Visualization of AI-driven clinical trials in mental disorders: study conditions, co-occurrence, and participant demographics. This figure summarizes key aspects of AI-driven clinical trials in mental disorders. (A) A Sankey diagram for the top 30 conditions investigated in these trials, illustrating the flow between conditions, study types, categories, and study statuses. (B) A heatmap of the top 20 conditions and their co-occurrence, using a logarithmic scale to highlight the relationships between common mental disorders. (C) Distribution of study statuses, emphasizing the number of ongoing, completed, and terminated studies. (D) Demographic information, including gender distribution and age distribution. These visualizations offer valuable insights into the scope, progress, and participant demographics of AI-based research in mental disorders. Created with Python (v3.10) with libraries such as Matplotlib and Seaborn. AI: Artificial intelligence.

**Figure 7A** illustrates the relationships across various dimensions, including conditions, research types, and research status, of clinical studies related to AI in mental disorders. The majority of these studies employ AI technologies for intervention or observation of mental disorders to evaluate the effectiveness of AI in early diagnosis, treatment optimization, and disease monitoring. However, a few studies have also integrated AI with other therapeutic technologies or treatments, suggesting that the combination of AI with other treatment modalities is a promising avenue for exploration in mental disorder research (Husnain et al., 2024; Olawade et al., 2024).

In investigations focused on specific diseases, our review found that numerous AI studies also evaluate diverse categories of mental disorders. These analytical approaches are frequently constructed to discriminate between multiple diagnostic classes or to detect co-occurring psychiatric phenotypes (Luján et al., 2022; Tutun et al., 2023). We also examined patterns of comorbidity across clinical trials. As shown in **Figure 7B**, displayed as a heatmap of the 20 most common comorbid mental disorders, depression frequently overlaps with multiple concurrent diagnoses in other disorders, especially cognitive impairment, Alzheimer’s pathology, sleep-wake dysregulation, and anxiety spectrum disorders. That is, depression-diagnosed patients often show additional neuropsychiatric comorbidities, highlighting the complex pathophysiology of depressive disorders and other clinical entities that share symptomatic manifestations (Arnaud et al., 2022). Cognitive dysfunction and Alzheimer’s disease exhibited significant comorbidity patterns, meaning that patients with cognitive decline often also show memory impairment or other neurodegenerative phenotypes, especially in older populations (Daiello and Tannenbaum, 2018). Anxiety-related conditions similarly exhibited significant diagnostic overlap with depressive disorders, Alzheimer’s pathology, and other mental disorders. These results provide additional support for the hypotheses that common symptomatic phenotypes and potential common pathogenic mechanisms exist across these disorders (Aslan, 2024; Carini and Seyhan, 2024). From a computational modeling perspective, comorbid presentations pose unique challenges for AI systems. Although there is considerable symptom overlap across disorders, most clinical data sets predominantly use single diagnostic labels to oversimplify conditions, thereby ignoring comorbid states that may blur classification boundaries and undermine predictive accuracy. Contemporary approaches include multi-label classification strategies that allow for multiple diagnoses to be assigned to a single patient, thereby improving clinical validity. Alternatively, multi-task learning architectures treat each diagnosis as a separate but related predictive task that is correlated across disorders for cross-disorder feature representation learning. Advanced computational strategies including attention-based neural networks and feature decomposition algorithms can improve the identification of disorder-specific biomarkers while reducing confounding effects from shared non-discriminative signals, thereby enhancing both model transparency and diagnostic accuracy.

**Figure 7C** is the current landscape of AI inclusion across clinical trials in mental disorders research based on study phases. The most of trials are currently in the recruiting phase (*n* = 174), reflecting growing momentum in the application of AI within psychiatric research. However, this also indicates that long-term clinical outcome data are still largely unavailable. A substantial number of studies have been completed (*n* = 146), offering multimodal datasets that can help assess the impact of AI-assisted tools on diagnostic accuracy, treatment adherence, and recovery trajectories. Meanwhile, a smaller group of trials remains in the preparatory phase (*n* = 60), and some have been terminated or withdrawn due to limitations in funding, ethical compliance, or methodological design. According to the gender and age distribution shown in **Figure 7D**, a significant majority (94.6%) of trials recruited both male and female participants, while 4.24% focused exclusively on women’s mental health, particularly conditions influenced by gender-related factors such as postpartum depression (Mousa et al., 2023). Only 1.16% of studies centered on male populations, typically involving groups like military personnel or individuals with PTSD. Adult participants comprised the largest subgroup (approximately 45%), consistent with the high prevalence of mental disorders in the adult population. Older adults (approximately 40%) were also quite numerous, particularly in trials focused on aging-related conditions like Alzheimer’s disease. Representation of children was much lower (approximately 15%) due to ethical limitations, complexity, and challenges inherent to pediatric mental health research (Salamanca-Buentello et al., 2020). Additionally, children also exhibit much more variability in their behavior patterns and typically require caregiver-reported assessments or observational data, which limits the availability of standardized, multimodal datasets. Pediatric mental health also presents several modeling challenges from a computational viewpoint: diagnostic criteria and expression of symptoms change with age, and therefore require age-aware architectures and training methods. Data collection in child-focused studies often involves modalities such as eye-tracking, audiovisual interactions, and gamified behavioral tasks, all of which are distinct from adult psychiatry. These differences highlight the need for multimodal integration and age-aware model design when developing AI systems for mental health across the lifespan. Moreover, they caution against overly broad generalizations when applying predictive models across different age groups.

**Figure 8:**
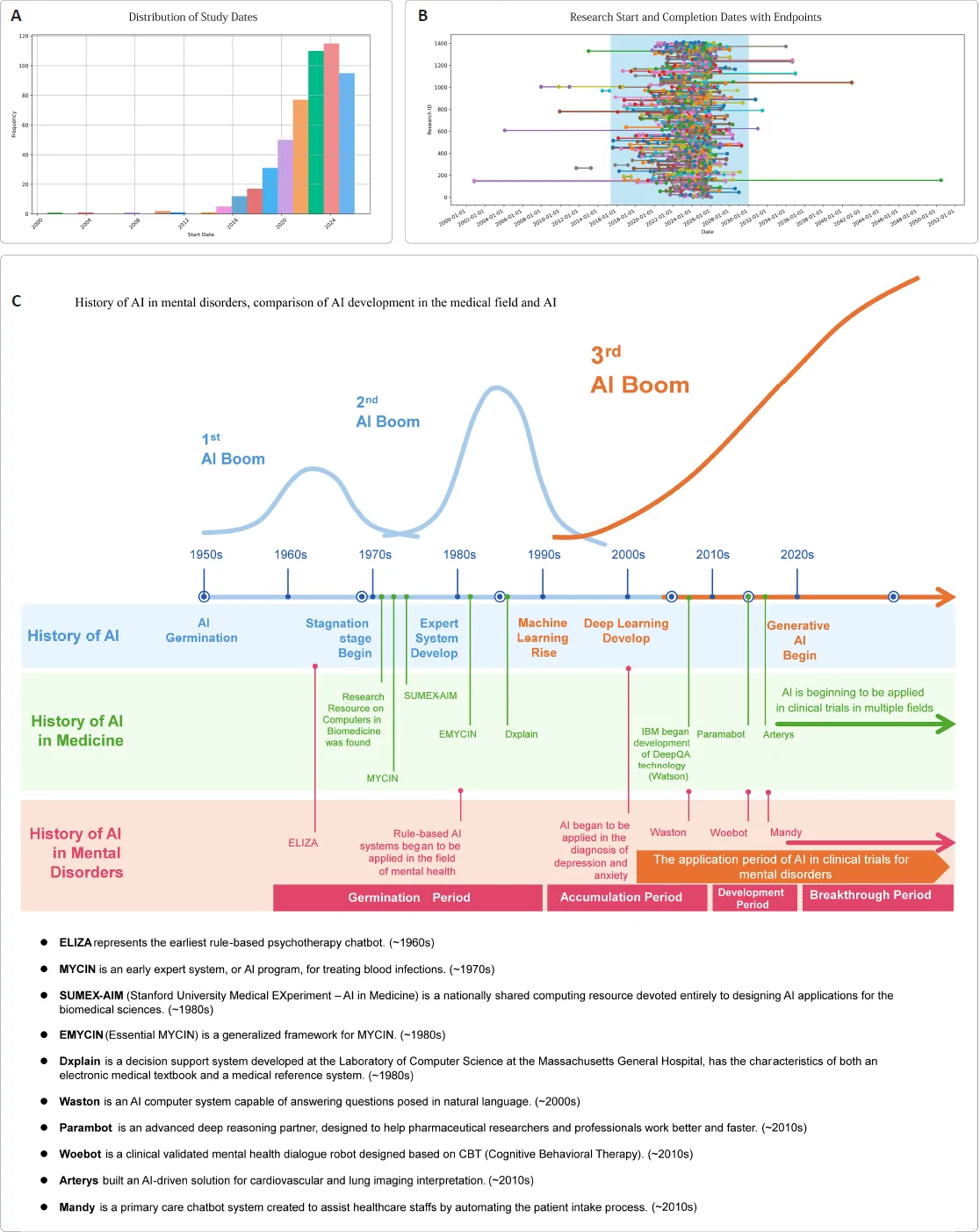
Timeline and development of AI in mental disorders: Research trends and milestones. This figure presents a comprehensive analysis of the history and development of AI in mental disorders, including research trends and key milestones. (A) Distribution of study start dates across different periods, illustrating the increasing trend of AI in mental disorder research over time. (B) Start times and durations of clinical trial studies, highlighting the progress and duration of various studies. The blue region marks the period with the highest concentration of research activity. (C) A detailed timeline comparing the history of AI in mental disorders with the broader history of AI development in the medical field. The timeline is divided into four distinct periods: the germination period (1960s–1990s), marked by the early use of AI in mental health; the accumulation period (1990s–2010s), characterized by the development of expert systems and machine learning; the development period (2010s–2020s), marked by the widespread use of deep learning technologies; and the breakthrough period (post-2020s), highlighting the emergence of generative AI and the initiation of clinical trials in mental disorders. This figure provides a clear overview of the significant advancements in AI technology and its growing role in mental disorder research and treatment. A and B were generated using Python (v3.10) with libraries such as Matplotlib and Seaborn. C was designed and assembled using Microsoft PowerPoint (v16.0). AI: Artificial intelligence.

Finally, an analysis of the start times and study durations shown in **Figure 8A** and **B** suggests that clinical practice involving AI in mental disorders has progressed from an initial exploratory stage to a stage of rapid growth. From a microscopic perspective, more and more studies are biased towards neuropsychiatric mechanisms and biomarker discovery. In the field of observational research, AI models are usually used to predict the structure, function, connection, and molecular characteristics of the brain before onset or before intervention. Feature engineering is mostly used to divide brain regions such as DLPFC, hippocampus, anterior cingulate, corpus callosum, and amygdala in more detail; Interventional research is more about using AI to predict and compare the structure, function, connection, and molecular characteristics of the brain before and after intervention. It is mainly based on the prediction results to explore the changes and mechanism of action of the brain during treatment. Currently, most studies focus on the prefrontal-limbic loop and thalamus. From a macro perspective, since 2016, there has been a marked increase in activity, with a particularly diverse range of studies initiated after 2020. Most of these trials are expected to conclude between 2024 and 2026, although a small number are projected to continue for several decades. Intervention studies typically span shorter timeframes, often less than 3 years, whereas observational studies tend to cover longer periods. This distribution reveals a structural limitation in the current research landscape. While short-duration trials are useful for evaluating technical metrics such as diagnostic accuracy and model performance, they do not allow for a meaningful assessment of long-term patient outcomes, such as relapse prevention, treatment adherence, or functional recovery. In addition, studies of extended duration remain rare or are still ongoing, and few provide follow-up data beyond the intervention phase. These gaps suggest that the long-term effectiveness of AI-based interventions in clinical psychiatry remains insufficiently validated, particularly with respect to sustained therapeutics. **Figure 8C** provides a clear comparison of the clinical research progress of AI in mental disorders with the broader development of AI in healthcare, tracing its origins back to the creation of the first chatbot, ELIZA (Weizenbaum, 1966), in 1966, which marked the early application of AI in psychological treatment. We designate from the 1960s to the 1990s as the embryonic phase of AI applications in mental disorders (Sun et al., 2023). The onset of the third wave of AI, driven by advances in ML and DL, and the development of AI in medical fields, particularly in clinical research, characterizes the period from the 1990s to the 2010s as the accumulation phase, during which AI applications were gradually expanded from other medical fields to mental disorders. The 2010s-2020s witnessed an explosive surge in research on AI in mental disorders and neuroscience, with research volumes increasing exponentially (Shimada, 2023), as indicated by the blue region in **Figure 8B**. Since the 2020s, the arrival of generative AI has marked a breakthrough phase in the application of AI in mental disorders (Tortora, 2024; Xian et al., 2024). At the same time, the issues that need to be addressed in parallel include privacy leaks, the mistakes caused by over-reliance on AI and AI delusion, as well as the moral and ethical concerns that cannot be ignored (Huang et al., 2023).

## Challenges and Prospects of Artificial Intelligence in Mental Disorders

### Navigating the hurdles: Ethical, technical, and operational challenges

In this emerging interdisciplinary research field, while many scholars have explored mental disorders, challenges for ML and DL persist, including data scarcity, lack of personalization, privacy issues, and interpretability. Despite rapid progress in AI applications for mental health, several critical challenges remain. These span technical limitations, ethical concerns about data privacy, and barriers to clinical adoption.

**Data issues:** Ongoing data issues in mental health research include data sparsity (limited data availability), data fragmentation (data coming from diverse sources), and data variation (inconsistent data types, such as neuroimaging, behavioral signals, and unstructured text). Due to the complexity of psychiatric disorders and diverse data types, data integration is also challenging. Usually, small data are available, and such small datasets increase the risk of overfitting and noise during training. Researchers attempted to use data augmentation methods, such as generative adversarial networks and diffusion models, to generate synthetic data to augment small datasets (Perez and Wang, 2017). Another strategy is transfer learning, where models are pretrained on large general medical datasets and fine-tuned on task-specific samples (Cheplygina et al., 2019). Additionally, regularization methods like dropout dynamically change neural network structures during training to help alleviate overfitting (Srivastava et al., 2014).

**Privacy concerns:** The diagnosis and personalized treatment of mental disorders involve highly private personal health information, such as psychological tests, treatment records, and current mental health status. Privacy concerns further increase when integrating more types of data, such as clinical records, social media posts, sensor outputs, and behavioral logs. These datasets often differ in term of ownership, structure, and regulatory protections, and their combination increases the risk of re-identification if proper privacy protection is not implemented. In general, matching across different modalities usually requires the existence of common identifiers or embedding linkage, which will all damage anonymity. Privacy-preserving methods, such as secure multiparty computation, differential privacy, and federated multimodal learning, have been proposed to address these issues (Lin et al., 2023). Among these, federated learning (McMahan et al., 2023) is a decentralized training approach where the training process of models runs on local devices, and thus raw data do not need to be transferred to a central server. This approach helps protect data privacy while allowing models to be customized on local devices. For example, Chen et al. (2020) proposed FedHealth, a federated learning framework to support personalized healthcare by aggregating heterogeneous data from multiple parties without privacy and security concerns.

**Interpretability:** The lack of transparency in DL models creates a barrier between computer scientists and biologists, a challenge that is particularly pronounced in LLMs. While these models offer high accuracy, they often fail to explain why those answers are correct. Their decision-making processes often appear opaque to clinicians and biologists. Developers should aim to design algorithms with simpler structures to balance interpretability and accuracy (Lo Piano, 2020). Additionally, KGs offer a relational perspective by connecting various types of information. Teng et al. (2020) advanced this with a method called G_Coder, using KGs to enhance the interpretability of medical code predictions. These studies collectively demonstrate the value of KGs in improving the efficiency and interpretability of disease diagnosis and treatment. Moreover, feature importance evaluation is also a way in which biological interpretability derived from neural mechanisms supports the interpretability of AI applications in mental illness.

Overall, addressing these challenges is critical to ensuring the safe, effective, and ethical integration of AI into mental healthcare systems.

### Unlocking the potential: Future directions and innovations

**Figure 9:**
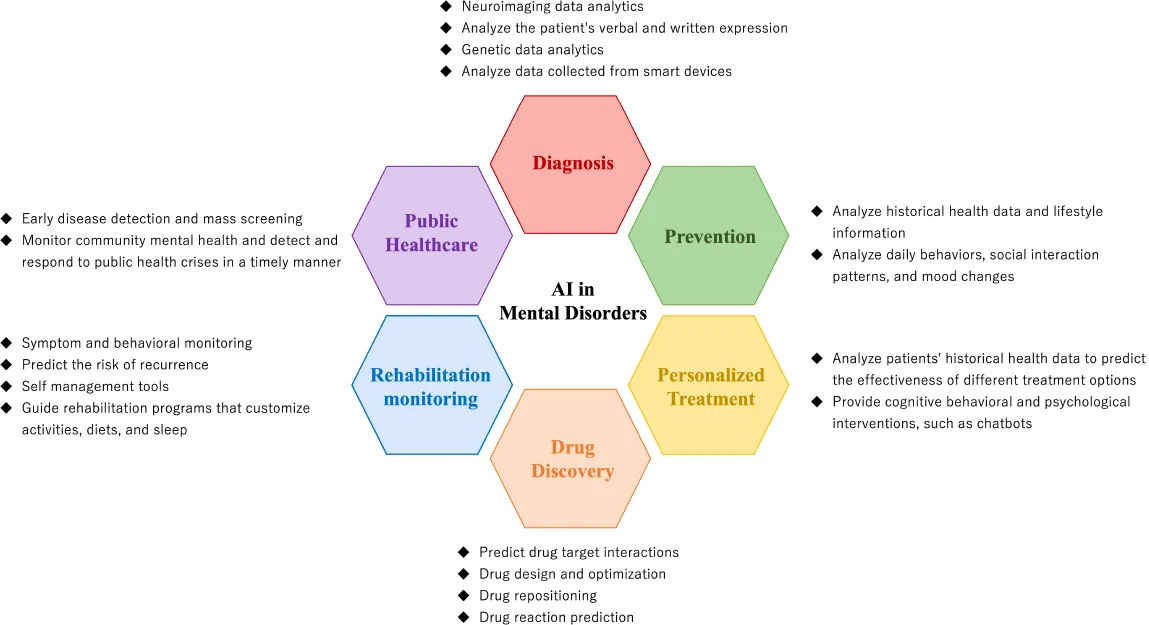
Future applications of AI in the field of mental disorders. This figure illustrates six key areas where AI demonstrates potential: (1) Prevention: leveraging historical health data and behavioral analysis to identify early signs of mental disorders and support lifestyle modifications; (2) Diagnosis: utilizing neuroimaging, genetic data, and patient interactions for accurate and timely mental health evaluations; (3) Personalized treatment: tailoring cognitive and behavioral interventions based on patient-specific health data; (4) Drug discovery: predicting drug-target interactions, optimizing drug design, and identifying potential drug repositioning opportunities; (5) Rehabilitation monitoring: tracking symptom progression and recurrence risk, and customizing rehabilitation plans for activities, diet, and sleep; and (6) Public health screening: enhancing community-level mental health surveillance to detect and respond to public health crises efficiently. AI: Artificial intelligence.

AI holds significant promise for advancing the diagnosis, stratification, and treatment of mental disorders. In **Figure 9**, we outline six core domains where AI innovation is likely to yield the greatest clinical benefit, ranging from early screening to real-time support (Lee et al., 2021).

One key opportunity lies in the development of multimodal and individualized diagnostic systems, capable of integrating behavioral, linguistic, physiological, and neuroimaging data. Recent advances in multimodal representation learning, including cross-modal attention, heterogeneous graph neural networks, and KGs, offer pathways to more nuanced and patient-centered modeling. These techniques may be especially important in handling comorbidity and the heterogeneous symptom structures characteristic of psychiatric illness (Zeng et al., 2022; Ma et al., 2023). The application of LLMs is also expanding rapidly in mental health. Beyond basic classification, LLMs such as MentaLLaMA, Mental-Alpaca, and domain-specific models like Chinese MentalBERT have shown promise in generating interpretable rationales, facilitating dialogue-based interventions, and enhancing contextual understanding. However, technical issues such as algorithmic bias, cultural and linguistic adaptation, and safety in sensitive applications still require careful attention.

Another underexplored area is the adaptation of AI for specific populations, including children, older adults, and individuals from diverse cultural backgrounds. These groups often exhibit unique behavioral or developmental patterns, requiring age- and context-sensitive models. Additionally, the current field focuses predominantly on short-term classification tasks. Future research should include longitudinal outcome modeling, tracking recovery trajectories, relapse risk, and social reintegration, which are key metrics in psychiatry (Graham et al., 2019; Yang et al., 2020; Ding and Hu, 2021; Feng et al., 2022).

## Limitations

First, the selected literature was only from PubMed, which means other literature from other databases or websites was not selected. This may lead to publication bias and language bias. Second, the KG was constructed based on the BioBERT-based method for NER. Although BioBERT has achieved relatively high performance, there may still be errors in NER, which may affect the accuracy of information extraction. Third, we enriched the application context by using clinical trial data; however, the latest data only came from 2021, and we did not use clinical data from the past three years. In addition, the KG and clinical data analysis in this review only reflect existing paradigms of AI applications in mental disorders. The possibility of using AI systems to independently assist in clinical decisions in psychiatric care has not been explored and deserves further attention.

## Conclusion

Our study offers a comprehensive examination of how AI techniques are being applied in mental disorder research, focusing on a structured KG comprising 3158 entities and 3248 relationships. By exploring the links between AI methodologies, categories of psychiatric conditions, and the types of data used, the work provides a nuanced overview of the methodological ecosystem shaping this field. Incorporating real-world clinical trial data, the study highlights current patterns in disease targets, research approaches, and patient populations involved in psychiatric AI research. Additionally, it situates the development of AI in mental health within a broader medical and technological context, drawing attention to specific domain challenges, implementation gaps, and areas for future advancement.

Despite remaining challenges, such as limited availability of longitudinal datasets, concerns over data privacy, cultural biases, and interpretability issues, ongoing developments in areas such as multimodal modeling, federated learning, and large-scale language models are opening promising pathways for more robust, transparent, and clinically relevant systems. Currently, in psychiatric practice, AI is more of a behind-the-scenes support than a front-line directive. There is still a large gap between the predictive models and their actionable clinical use. The gap between functional integration and model proliferation should receive more attention than just counting the number of studies or systems.

In summary, this work paves the way for future advancements in AI-assisted precision psychiatry. We believe this work contributes toward more personalized, ethical, and clinically useful care for mental health. In the future, we will extend the work with more diverse and representative clinical data and connect the KG with real-world medical data repositories. We also plan to evolve the KG into a more flexible and patient-centered decision support system. In parallel, we will develop a recommendation engine extracted from the KG such that appropriate AI models can be recommended for different psychiatric diagnoses and clinical scenarios. We expect these two directions can bridge the gap between more abstract research findings and more clinical applications.

## Additional files:

***Additional Table 1:*** *Selection criteria for literature mining keyword categories.*

***Additional Table 2:*** *Summary of AI in early detection and diagnosis of mental disorders.*

***Additional Table 3:*** *Summary of AI in personalized treatment and care for mental disorders.*

## Data Availability

*All relevant data are within the paper and its Additional files.*
